# Cross-alteration of murine skin and tick microbiome concomitant with pathogen transmission after *Ixodes ricinus* bite

**DOI:** 10.1186/s40168-023-01696-7

**Published:** 2023-11-11

**Authors:** Nathalie Boulanger, Jean-Louis-Marie Insonere, Sebastian Van Blerk, Cathy Barthel, Céline Serres, Olivier Rais, Alain Roulet, Florence Servant, Olivier Duron, Benjamin Lelouvier

**Affiliations:** 1 UR7290: Virulence bactérienne précoce: groupe Borrelia, FMTS, University of Strasbourg, Strasbourg, France; 2 Vaiomer, 516 rue Pierre et Marie Curie, 31670 Labège, France; 3 Laboratoire d’écologie et d’épidémiologie parasitaires Institut de Biologie, University of Neuchatel, 2000 Neuchâtel, Switzerland; 4 Maladies Infectieuses et Vecteurs: Ecologie, Génétique, Evolution et Contrôle (MIVEGEC), Centre National de la Recherche Scientifique (CNRS), Institut pour la Recherche et le Développement (IRD), Université de Montpellier (UM), 911 Avenue Agropolis, 34394 Montpellier, France

**Keywords:** Microbiota, Metagenome, Lyme disease, *Borrelia*, *Borreliella*, *Anaplasma*, 16S targeted sequencing, Metagenomic

## Abstract

**Background:**

Ticks are major vectors of diseases affecting humans such as Lyme disease or domestic animals such as anaplasmosis. Cross-alteration of the vertebrate host skin microbiome and the tick microbiome may be essential during the process of tick feeding and for the mechanism of pathogen transmission. However, it has been poorly investigated.

**Methods:**

We used mice bitten by field-collected ticks (nymphs and adult ticks) in different experimental conditions to investigate, by 16S rRNA gene metabarcoding, the impact of blood feeding on both the mouse skin microbiome and the tick microbiome. We also investigated by PCR and 16S rRNA gene metabarcoding, the diversity of microorganisms transmitted to the host during the process of tick bite at the skin interface and the dissemination of the pathogen in host tissues (blood, heart, and spleen).

**Results:**

Most of the commensal bacteria present in the skin of control mice were replaced during the blood-feeding process by bacteria originating from the ticks. The microbiome of the ticks was also impacted by the blood feeding. Several pathogens including tick-borne pathogens (*Borrelia/Borreliella*, *Anaplasma*, *Neoehrlichia*, *Rickettsia*) and opportunistic bacteria (*Williamsia*) were transmitted to the skin microbiome and some of them disseminated to the blood or spleen of the mice. In the different experiments of this study, skin microbiome alteration and *Borrelia/Borreliella* transmission were different depending on the tick stages (nymphs or adult female ticks).

**Conclusions:**

Host skin microbiome at the bite site was deeply impacted by the tick bite, to an extent which suggests a role in the tick feeding, in the pathogen transmission, and a potentially important impact on the skin physiopathology. The diversified taxonomic profiles of the tick microbiome were also modified by the blood feeding.

Video Abstract

**Supplementary Information:**

The online version contains supplementary material available at 10.1186/s40168-023-01696-7.

## Introduction

Arthropod-borne diseases have long been considered as a three-actor system with complex interactions involving the vector, a single pathogen, and the vertebrate host. More recently, the microbiome of both the vector and the vertebrate host has been proposed as a potential key driver of this system, regulating the transmission of potential pathogens from the vector to the host [1–3]. However, the role of the vertebrate host microbiome in pathogen transmission, especially at the skin interface, has been poorly investigated so far [4]. Besides, within the vector, the gut and salivary gland microbiomes modulate pathogen infection possibly through a subtle regulation of the arthropod immune system [1]. This aspect has been particularly studied in mosquitoes [5], and the role of mosquito microbiome is now investigated as a potential new strategy of disease control [6, 7].

Ticks are major vectors of diseases affecting humans such as Lyme disease and domestic animals, such as anaplasmosis [8–10]. The genus *Ixodes* is of particular importance as a vector for these major pathogens in the northern temperate hemisphere [11–13]. Hard ticks, such as *Ixodes*, are exclusively hematophagous ectoparasites and undergo four life stages: eggs, larvae, nymphs, and adults. Between each stage, they feed for several days, but they spend most of their time questing on vegetation and rehydrating in the leaf litter. Members of the *I. ricinus* complex can feed on a wide diversity of hosts including humans, domestic and wild mammals, birds, and reptiles. These ticks are therefore exposed to a great variety of microorganisms and various host microbiomes during their life cycle [14–16]. The tick microbiome attracts more and more scientists’ interest as a potential regulator of pathogen development and transmission [1, 17]. Early and more recent bacterial 16S rRNA gene metabarcoding (16S rDNA targeted next-generation sequencing) identified more than a hundred bacterial genera in the different tick stages of members of the *I. ricinus* complex [18–20]. Variations in tick microbiome were found according to geography and environment [19–22]. The main phyla composing the tick microbiome are usually *Proteobacteria*, and to a lesser extent, *Firmicutes*, *Actinobacteria*, and *Bacteroidetes* [18–20, 23, 24]. Most of these bacteria are external microbes inhabiting tick cuticles, while the diversity of internal bacteria is usually lower [22, 23]. The internal microbes include diverse tick-borne pathogens, but also non-pathogenic and often mutualistic endosymbionts [16, 25]. Some of these endosymbionts have a key nutritional function for ticks, such as providers of B vitamins [25, 26]. The bacterium Canditatus *Midichloria* is an intramitochondrial endosymbiont [27] commonly detected in *I. ricinus* [19, 20, 28, 29], and it is usually assumed that it is the nutritional endosymbiont providing B vitamin to this tick species [25]. However, very few studies have started to investigate the role of arthropod microbiome during the process of tick bite and particularly during pathogen transmission in tick-borne diseases [22, 30]. On the other hand, the role of the vertebrate microbiome is increasingly being investigated as a regulator of various physiological or pathological processes [31]. In the context of vector-borne diseases, the microbiome is little or not explored and when it is, it is mainly the vector microbiome that is studied, but independently of the skin microbiome of the vertebrate host. However, it is very likely that the microbiome of the vector and the one of the vertebrate host have a combined effect at the skin interface during the transmission of pathogens.

The process of pathogen transmission by hard ticks generally takes place within the first 24 h of the blood meal and it increases with time [32]. The potential co-transmission of tick microbiome and other impacts of tick bite to the host skin microbiome during the long-lasting blood meal has never been investigated so far. In insects, the sandfly microbiome was shown to impact the development of infection during the transmission of *Leishmania* parasite to the vertebrate host by triggering the inflammasome [2, 33].

Overall, while the tick microbiome, on the one hand, and the host skin microbiome on the other hand are increasingly investigated, the interaction of the two microbiomes during the process of blood feeding and its role in the horizontal pathogen transmission is so far insufficiently studied. This interaction could induce a cross-alteration of both microbiomes with a potentially crucial role in tick-borne diseases as hard ticks remain attached to the skin of the host for several days.

Advances in DNA sequencing technology have greatly improved the study of microbiomes and the identification of pathogens. We previously set up a contamination-aware approach to specifically study low biomass samples such as blood, skin, and tissue microbiome, despite the challenges associated with those sample types (low bacterial DNA quantity impacted by technical and environmental bacterial DNA contamination and presence of a high amount of PCR inhibitors in the sample) [34–37]. We use this technology to investigate on mouse models the diversity of microorganisms transmitted during the process of tick bite and the overall impact of tick bite on the skin microbiome of the vertebrate host. We collected *I. ricinus* in the field and fed them during various days on laboratory mice. We collected the skin at the site of the tick bite as well as different organs (spleen and heart) and blood to measure potential bacteria transmission and dissemination and assess the impact of the tick bite on the commensal skin microbiome of the mice. We also analyzed field-collected nymph and adult ticks (unfed or fed on the mice). We used 16S metagenomic targeted sequencing to study the mouse skin microbiome and the tick microbiome alterations, the transmission kinetics, and dissemination of tick-borne pathogenic bacteria such as *Borrelia* and *Anaplasma* genera.

## Material and methods

### Mice and ticks

The C3H/HeN mice were obtained from Charles River and maintained in the animal facility, by 2 to 5 per cage with bedding before the experiments (Institute of Bacteriology, University of Strasbourg, France). For the experiments, we used males or females, 2–3 months old, maintained individually in a cage without bedding. Mice were kept at a temperature of 22/24°C, with a humidity level of around 50%. Lighting programming (day/night) was automated 12h/12h, with a 45-min dimming period at sunrise and sunset. They were housed in animal cabinets that were directly connected to the animal house air handling units. They were checked twice a day to check for potential problems in the course of the experiments. No behavior or health problems were noticed.

The protocols carried out in this study were approved by the Comité Régional d’Ethique en Matière d’Expérimentation Animale de Strasbourg (CREMEAS—Committee on the Ethics of Animal Experiments of the University of Strasbourg). Reference of the ethics statement is /No. CREMEAS 2020011416399363/APAFIS #23601. The protocols performed on animals follow the European guidelines: “directive 2010/63/EU” under the animal facilities #: d67-482-34. *Ixodes ricinus* ticks were collected on vegetation during the seasonal activity peak (March-June) in different sites around Strasbourg (Alsace - Est of France), a highly endemic area for tick-borne diseases. These sites are known to be endemic for *B. burgdorferi* sensu lato, *A. phagocytophilum*, *Borrelia miyamotoi*, and potentially other microorganisms [38]. Adult ticks and nymphs were collected at the same time and placed alive in different tubes. To ensure that the ticks used in the experiment belonged to *Ixodes ricinus* species, in addition to morphological identification, tick batches were regularly checked by MALDI-TOF mass spectrometry as described previously [39]. Until the feeding of mice, ticks were maintained alive in a humid chamber with 80 % humidity at room temperature and a 12h/12h photoperiod.

### Protocols of microorganism transmission and dissemination

In the case of *I. ricinus* ticks, the transmission of bacterial pathogens to the host skin is known to start at the bite site within the first 24 h of the blood meal [15]. Once the bacteria pathogens are in the skin, bacterial multiplication and transmission to other body parts occur (with a peak around day 10 for *Borreliella*) [15, 40]. To assess transmission in the first days of the blood meal, we removed the ticks and collected the skin itself after 1 to 4 days of blood meal. To assess the dissemination of the pathogens in other organs, we removed the ticks and kept the mice without ticks for an additional 10 days to let the pathogens amplify and disseminate from the skin to the other organs

**Fig. 1:**
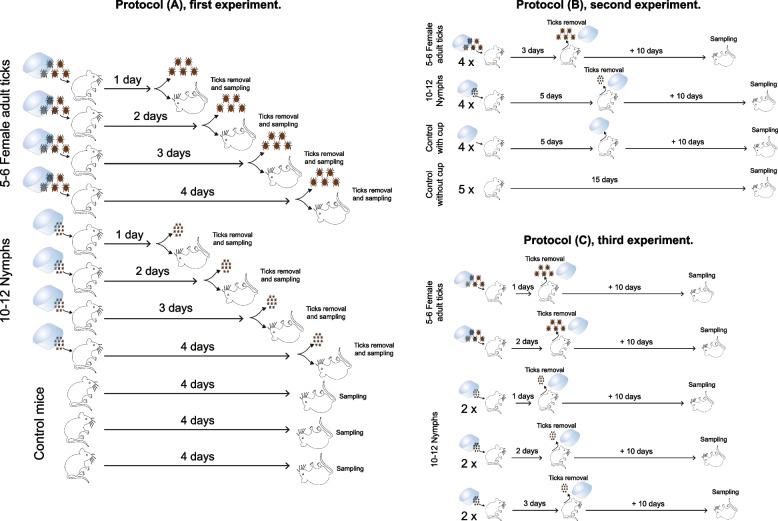
Description of the three different protocols used in different experiments

Three different experiences (transmission or dissemination protocol) were performed in our study as detailed below and in Fig. 1.

**Fig. 2:**
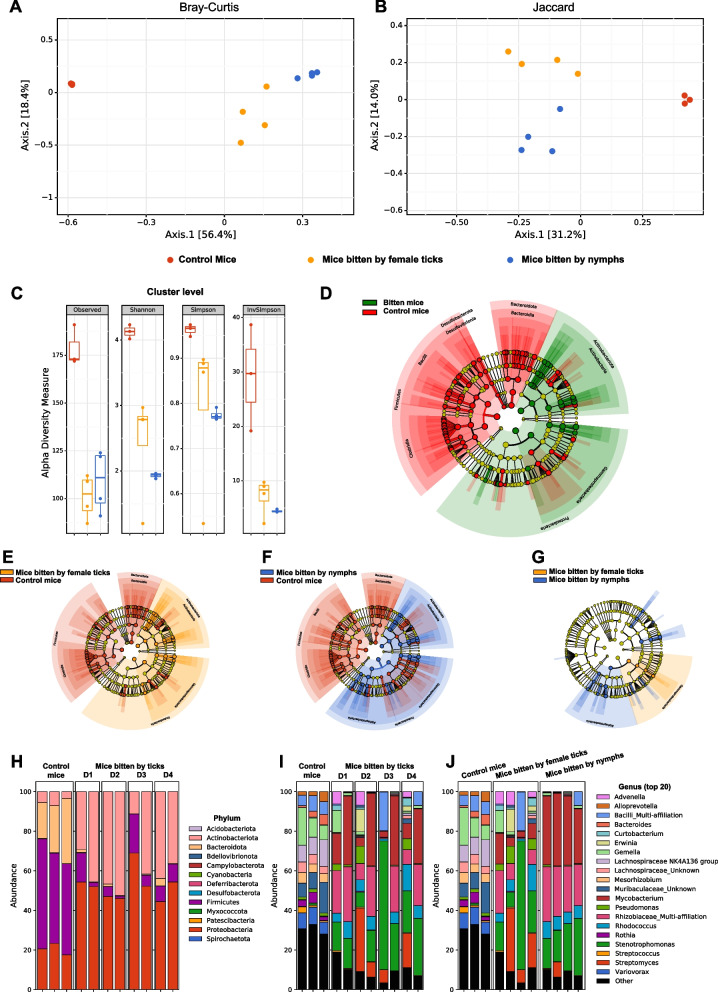
Skin microbiomes from mice bitten by nymphs or adult female ticks are deeply and differently modified. **A**, **B** Beta diversity ordination (PCoA) using Bray Curtis (**A**) and Jaccard (**B**) distances of the skin microbiome of control mice (red dots), mice bitten by female adult ticks (yellow dots), and mice bitten by nymphs (blue dot) for 1 to 4 days. **C** Alpha diversity (cluster levels observed, Shannon, Simpson, and inverse Simpson indexes) of the same skin samples. **D**–**G** LEfSe (linear discriminant analysis effect size) analysis summarizing the taxa significantly modified (*p*<0.05 with Mann-Whitney nonparametric test) between the skin of control mice and bitten mice (by nymphs or female ticks) (**D**), control mice and mice bitten by female ticks (**E**), control mice and mice bitten by nymphs (**F**), and mice bitten by female ticks and mice bitten by nymphs (**G**). **H–J** Barplots of relative proportions in the skin biopsies of the phyla (**H**) and top 20 genera (**I** and **J**), grouped by duration of feeding (**H** and **I**) or by tick stages (**J**)

Protocol (A), first experiment (Fig. 2, Tables 1 and 2). In the first study of microorganism transmission from ticks, either a pool of 10 to 12 *I. ricinus* nymphs or a pool of 5 or 6 female adult *I. ricinus* were fed on each mouse according to Mbow et al. [41]. Briefly, the mouse skin on the back was shaved and a plastic cup was glued with wax. Ticks were introduced without prior washing of the cuticle, within the cup, and sealed with tissue tape. Each mouse was maintained individually in a cage without bedding. Ticks were removed on days 1, 2, 3, and 4, and mice were directly euthanized by cervical dislocation after tick removal. Mice were then dissected under a clean laminar flow under sterile conditions. First, the blood was collected, then the heart, the spleen, and the skin were sampled. The mouse skin was collected at the bite site after euthanasia. The entire bite area (between 20 and 180 mm^2^ of the skin, epidermis and dermis) was sampled using sterile forceps and scissors. All instruments were changed between each animal. The control consisted of the skin from mice not bitten by ticks, either just shaved in the back or shaved and set up with the plastic cup.

**Table 1:**
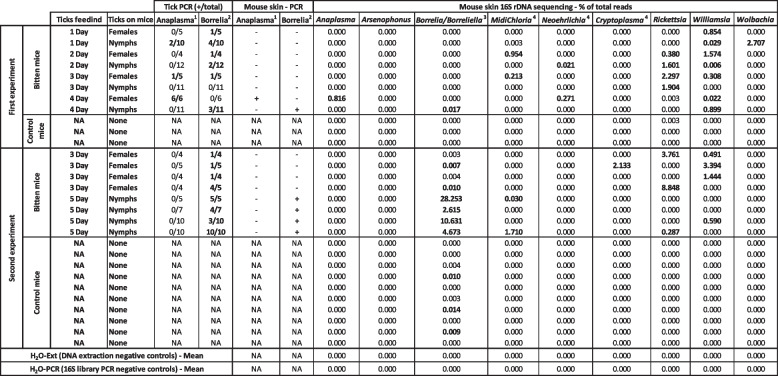
Detection by PCR and 16S rDNA sequencing of bacterial pathogens and bacteria of interest in the mouse skin of experiments 1 and 2

*Anaplasma* and *Borrelia burgdorferi sensu lato* were also tested by PCR in the ticks in contact with the corresponding mice (number of positive ticks in PCR/total tested) and in the mouse skin (**+** positive in PCR; - negative in PCR). *NA* not tested (control mice without ticks); *H*_*2*_*O-Ext* 10 tubes of molecular grade water extracted, amplified, and sequenced at the same time as the samples; *H*_*2*_*O-PCR* 12 tubes of molecular grade water amplified and sequenced at the same time as the extracted DNA of the samples

**Table 2:**
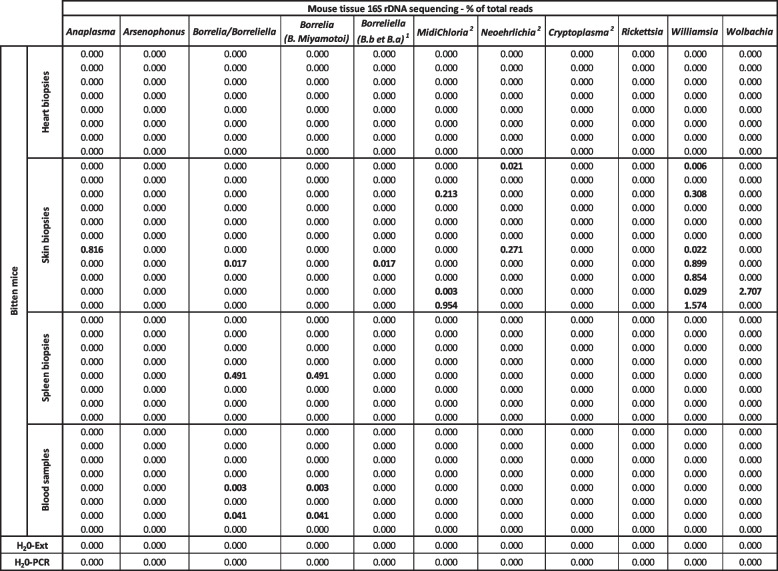
Detection of bacteria of interest by 16S rDNA sequencing in different tissues of the mice of experiment 1

*H*_*2*_*O-Ext* 10 tubes of molecular grade water extracted, amplified, and sequenced at the same time as the samples; *H*_*2*_*O-PCR* 13 tubes of molecular grade water amplified and sequenced at the same time as the extracted DNA of the samples

**Fig. 3:**
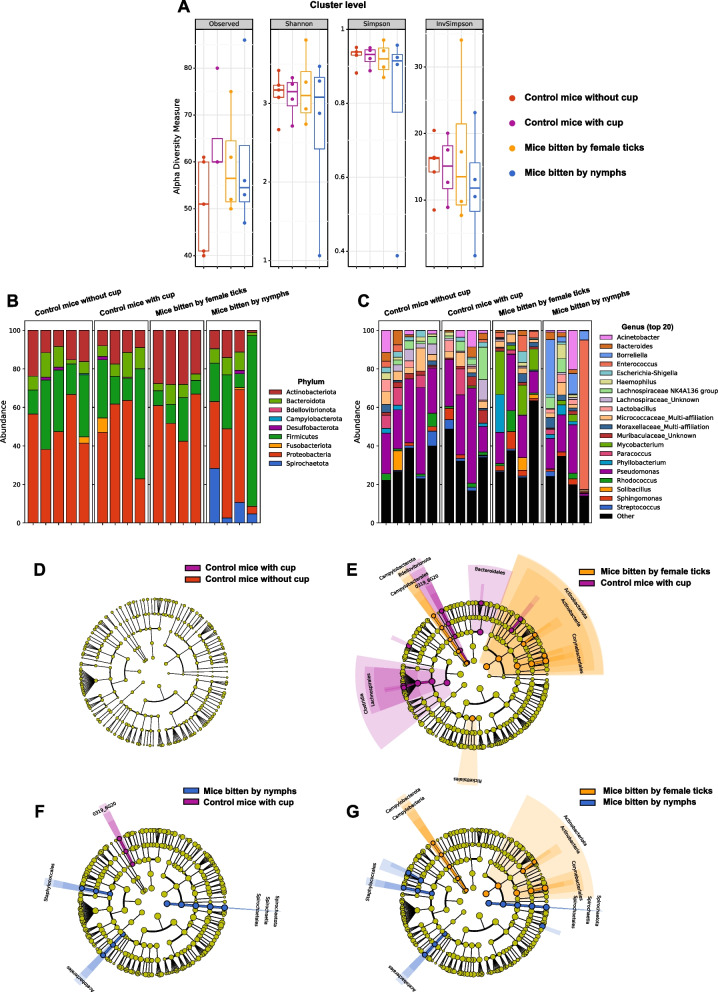
Impacts on the mouse skin microbiome persist 10 days after the removal of the ticks. **A** Alpha diversity (cluster levels observed, Shannon, Simpson, and inverse Simpson indexes) of the skin microbiome of control mice without a cup (red dots), control mice with a cup (purple dots), mice bitten 3 days by female ticks (yellow dots), and mice bitten 5 days by nymphs (blue dot). Bitten mice were kept 10 days after the removal of the ticks, before being euthanized. **B**, **C** Barplots of relative proportions in the skin biopsies of the phyla (**B**) and top 20 genera (**C**). **D**–**G** LEfSe (linear discriminant analysis effect size) analysis summarizing the taxa significantly modified (*p*<0.05 with Mann-Whitney nonparametric test) between skin of control mice without cup and control mice with a cup (**D**), control mice with a cup and mice bitten by female ticks (**E**), control mice with a cup and mice bitten by nymphs (**F**), and mice bitten by female ticks and mice bitten by nymphs (**G**)

Protocol (B), second experiment (Fig. 3, Table 1). In the protocol of microorganism dissemination in the mouse, either a pool of 10 to 12 *I. ricinus* nymphs or a pool of 5 or 6 female adults *I. ricinus* were fed using the same protocol described in (A). For female ticks, the plastic cup was removed and ticks were collected from the mice on day 3 to avoid potential high anemia induced after a complete blood meal by female adult ticks (circa 10 days). For nymphs, the plastic cup was removed and the nymphs were collected from the mice on day 5 (complete blood meal). Then, mice were maintained individually in cages with bedding for an additional 10 days. After 10 days, mice were euthanized and the blood, the heart, the spleen, and the skin biopsy were sampled as described above In this second experiment, to investigate more precisely, the potential role of the plastic cup on the mouse microbiome composition, additional negative controls were included: (1) mice shaved without a cup and maintained without bedding and (2) mice shaved with a cup and sealed with tape.

**Fig. 4:**
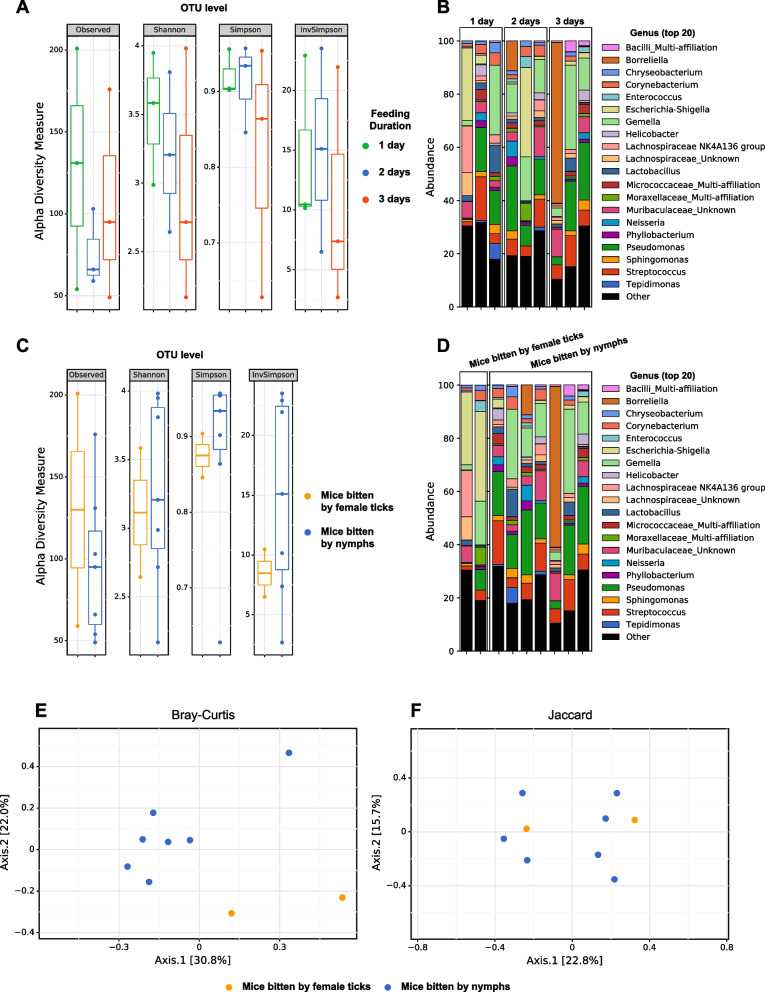
Long-term skin microbiome modifications are different between mice bitten by female ticks or by nymphs. **A** Alpha diversity (cluster levels observed, Shannon, Simpson, and inverse Simpson indexes) of the skin microbiome of mice 10 days after a blood feeding by tick (female ticks or nymphs) of 1 day (green dots), 2 days (blue dots), or 3 days (red dot). **B** Barplots of relative proportions in the skin biopsies of the top 20 genera in the samples separated by duration of the feeding. **C** Alpha diversity (cluster levels observed, Shannon, Simpson, and inverse Simpson indexes) of the same samples separated between mice bitten by female ticks (yellow dots) or by nymphs (blue dots). **D** Barplots of relative proportions in the skin biopsies of the top 20 genera in the samples separated by tick stages. **E**,** F** Beta diversity ordination (PCoA) using Bray Curtis (**E**) and Jaccard (**F**) distances of the skin microbiome of mice bitten by female ticks (yellow dots) or by nymphs (blue dots)

Protocol (C), third experiment (Fig. 4, Table 3). A third experiment was set up, to measure more precisely the process of transmission and dissemination of bacteria. Ticks (a pool of 10 to 12 nymphs or a pool of 5 or 6 females) were fed on mice during either 1, 2, or 3 days using a protocol similar to (A) and (B), then ticks were removed and mice maintained 10 additional days, individually in the cage with bedding, before being euthanized and sampled at day 11, day 12, and day 13, respectively, as described above. During the removal of the ticks from the mice, for one mouse, two unintended nymphs were found in addition to the 5 females of the mouse planned to be bitten only by 5 females. Consequently, we excluded this mouse from further analysis.

**Table 3:**
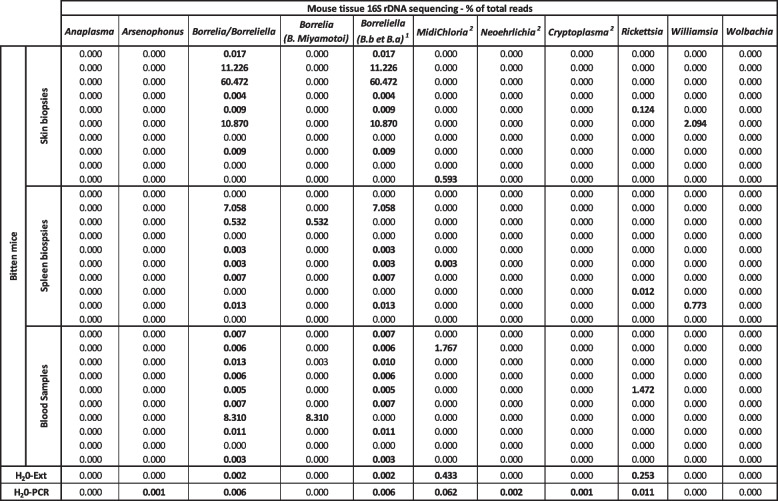
Detection of bacteria of interest by 16S rDNA sequencing in different tissues of the mice of experiment 3

*H*_*2*_*O-Ext* 30 tubes of molecular grade water extracted, amplified, and sequenced at the same time as the samples; *H*_*2*_*O-PCR* 20 tubes of molecular grade water amplified and sequenced at the same time as the extracted DNA of the samples

For each experiment, most of the ticks of the pool (5 to 12 for the nymphs, 4 to 6 for the female ticks) bit the mice for the planned duration of the blood feeding. Few ticks did not attach or died of dehydration before the bite.

To analyze the ticks in PCR (protocols A and B) or 16S metabarcoding (protocol C), each tick of the pool that fed on the mice until the end of the experiment was recovered and analyzed individually.

Ticks were not washed before microbiome analysis.

### Specific PCR targeting Borrelia burgdorferi sensu lato or Anaplasma phagocytophilum in mouse skin

DNA was extracted from the mouse skin or from ticks using the MagNA Pure system as described by the manufacturer (Roche Diagnostics, Switzerland). For *B. burgdorferi* sensu lato detection, the PCR targeted the *flagellin B* gene as described [40]. *A. phagocytophilum* was detected with a real-time PCR assay targeting the *msp2/p44* gene on the ABI7500 PCR apparatus (Applied Biosystems) as previously described [42].

### 16S rRNA gene amplicon sequencing (16S metabarcoding)

#### DNA extraction and sequencing library construction

DNA from 5.2 to 170.4 mg of the skin biopsy, 50 µl of whole blood samples, 115.5 to 195.3 mg of the heart, 2.4 to 10.1 mg of the spleen or individual whole ticks were extracted and amplified in a strictly controlled environment at Vaiomer (Labège, France) using a stringent contamination-aware approach as described previously [34–37].

Library preparation was performed by two-step PCR amplification using 16S universal primers targeting the V3–V4 region of the bacterial 16S ribosomal RNA gene (rDNA) as described previously [34]. The resulting amplicon of approximately 467 base pairs was sequenced using 2 x 300 paired-end MiSeq kit V3. For each sample, a sequencing library was generated by the addition of sequencing adapters. The detection of the sequencing fragments was performed using the MiSeq Illumina technology.

#### Bioinformatic analysis of the 16S rRNA gene sequencing data

The targeted metagenomic sequences were analyzed using the bioinformatic pipeline established by Vaiomer based on the FROGS (Find, Rapidly, OTUs with Galaxy Solution) guidelines [43]. Briefly, after demultiplexing of the barcoded Illumina paired reads, single-read sequences were cleaned and paired for each sample independently into longer fragments. Single-linkage taxonomic clusters (named “clusters” in the rest of the manuscript) were produced via single linkage clustering using the Swarm algorithm and its adaptive sequence agglomeration [43]. The taxonomic assignment was performed against the Silva v138.1 database to determine taxonomic profiles. To be noted that in the Silva 138.1 database, Lyme-associated *Borrelia* species (such as *B. burgdorferi* s.s. and *B. afzelii*) are grouped into the *Borreliella* genus whereas *Borrelia* species from the relapsing fever group, such as *B. miyamotoi*, belong to the Borrelia genus [44]. In this study, 16S sequences assigned to *Candidatus* Cryptoplasma genus in the Silva 138.1 database were noted as assigned to *Candidatus* Allocryptoplasma (correct current name).

The following specific filters were applied for this analysis to obtain the best results: (1) the last 40 bases of reads R1 were removed, (2) the last 40 bases of reads R2 were removed, (3) amplicons with a length of <350 or >500 nucleotides were removed, and (4) clusters with abundance <0.005% of the whole dataset abundance were removed.

Sequencing depth (number of clustered sequences per sample after filters) was on average 33,300 sequences. There was no significant difference in sequencing depth (Kruskal-Wallis test) between the samples of the different groups of analysis; thus, no transformation nor rarefaction of the data was needed.

### Statistical analyses

To analyze the differences in terms of alpha diversity and taxonomic composition, nonparametric tests were used: Mann-Whitney’s test was used when two groups were compared, Kruskal-Wallis test followed by a post hoc test (Dunn’s/Wilcoxon test) was used when more than two group settings. LEfSe analysis illustrated the results of Mann-Whitney’s test on the two group settings. Beta diversity differences were assessed using PERMANOVA (permutational analysis of variance) test to study the differences between sample groups in terms of centroid and/or dispersion [45], and using PERMDISP (multivariate dispersion) test to study the dispersion alone [46]. For upset plot and Venn diagram representation, a pre-filtering of structural zero using ANCOM II preprocessing method was applied to reduce the false discovery rate of zero-inflated data [47].

### Negative controls

To ensure a low background signal from bacterial contamination present in reagents and consumables, negative controls consisting of molecular grade water were added in an empty tube separately at the DNA extraction and at the PCR steps and amplified and sequenced concomitantly to the samples. Beta diversity analyses (Supplemental Fig. 1A, B) show a clear separation between the respective extraction negative controls and both tick samples (*p*<0.0005 with a PERMANOVA test, *n.s.* with a PERMDISP test using Generalized UniFrac alpha = 0.4) and skin samples (*p*<0.0075 with a PERMANOVA test, *n.s.* with a PERMDISP test using Generalized UniFrac alpha = 0.4). In addition, Venn diagrams of the common clusters present in microbiomes of the ticks (Supplemental Fig. 1C) or the mouse skin biopsies (Supplemental Fig. 1D) and their respective negative controls (H2O-Ext) show that there are only few clusters in common: 97.1% of the clusters found in ticks and 90.0 % of the clusters found in skin biopsies are not present in the negative controls. Finally, relative proportions in the negative controls of each taxon of interest studied in the skin, blood, heart, and spleen are displayed with the results in Tables 1, 2 and 3. All these controls confirm that bacterial contamination was well contained in our pipeline and had no impact on the results of this study as published beforehand [34–36].

## Results

### Skin microbiome from mice bitten by nymph or adult female ticks is altered

The study involved exposing mice to the bites of field-collected nymphs and adult female ticks. The microbiome of the mouse skin biopsies (sampled at the biting site) was characterized by 16S rRNA gene bacterial metabarcoding and compared to skin biopsies from control mice (Fig. 2). Beta diversity PCoA ordinations using Bray Curtis (Fig. 2A) and jaccard (Fig. 2A) distances show that the skin microbiome from bitten mice is well differentiated from the skin microbiome of control mice (*p*<0.0005 with a PERMANOVA test, *p*=0.03 with a PERMDISP test for both Bray-Curtis and Jaccard distances). In addition, beta diversity differs between skin biopsies of mice bitten either by nymphs or adult female ticks (Fig. 2A, B, *p*=0.03 with PERMANOVA; *p*=0.03 with PERMDISP for both Bray-Curtis and Jaccard distances).

Alpha diversity analyses (cluster levels observed, Shannon, Simpson, Inverse Simpson indexes) show a dramatic decrease in the skin microbiome of mice bitten by ticks versus controls (*p*=0.0143 in Mann Whitney test between the three controls and the eight bitten mice) (Fig. 2C)*.* The alpha diversity of different mouse skins bitten by nymphs showed similar levels in Shannon’s index but differed from control mice (*p*=0.03 with Kruskal-Wallis and Wilcoxon rank sum post hoc tests) and mice bitten by females (*p*=0.02 with Kruskal-Wallis and Wilcoxon rank sum post hoc tests).

LEfSe analyses (linear discriminant analysis effect size) show dramatic changes in the microbiome composition between skin of the three control mice and skin of the mice bitten by ticks (nymphs or adults) (Fig. 2D), between the three controls and the mice bitten by female ticks (Fig. 2E) between the three controls and the mice bitten by nymphs (Fig. 2F) and between mice bitten by females and bitten by nymphs (Fig. 2G). The majority of bacterial taxa, at all phylogenic levels, were either significantly increased or decreased between the different groups of mice.

Barplots of relative proportions of the top 20 taxa (Fig. 2H–J), or all taxa over 1% of the reads (Supplemental Fig. 2) confirmed the dramatic impact of tick bite on the mice skin microbiome, from phylum levels (Fig. 2H) to genus level (Fig. 2I-J and Supplemental Fig. 2). At the phylum level (Fig. 2H), during the tick bites (both adult ticks and nymphs), a striking decrease of Firmicutes and Bacteroidota (which are the main phyla in the skin of control mice) was observed while Proteobacteria and Actinobacteria increased significantly, independent of the duration of the blood meal, from 1 to 4 days. Analysis at the genus level (Fig.  2I-J and Supplemental Fig. 2) confirmed the impact of tick bite, and it illustrated that almost all the genera present in the skin of control mice were altered or replaced by other genera in the skin of mice bitten by ticks. From phylum to cluster levels, 321 taxa were significantly different between the three control mice and skin of mice bitten by ticks (nymphs or females) as illustrated in Fig. 2D and the barplot in Fig. 2H (phylum) and Fig. 2I-J (genus). Most of the bacteria from the control mice microbiome were replaced by other bacteria in mice bitten by ticks (females or nymphs). For instance, at the genus level, genera from Lachnospiraceae family, genera from Muribaculaceae family, *Mesorhizobium* genus, *Rothia* genus, and *Variovorax* genus virtually disappeared from the skin microbiome in mice bitten by ticks. Conversely, *Advenella*, *Erwinia*, *Mycobacterium,* genera from Rhyzobiaceae, *Rhodococcus*, *Stenotrophomonas*, *Streptomyces* appear in skin biopsies of mice bitten by ticks and constitute most of their microbiome. In addition, taxa such as *Mycobacterium* genus, Rhizobiaceae family were more present in skin biopsies of mice bitten by nymphs than skin biopsies of mice bitten by female adults.

Barplots grouped by days of feeding (Fig. 2H-I and Supplemental Figure 2) show that there were not many differences between days of feeding. The impact of the tick bites on the skin microbiome was already clear after one day of feeding by both adult ticks and nymphs and did not further change significantly over the duration of the feeding. However, barplots grouped by tick maturity (Fig. 2J) showed a clear difference between the microbiome of mice bitten by adult ticks or nymphs. Unlike control mice, there were many common genera between the mice bitten by adult ticks or nymphs, but the proportion of the genera differed between these two mouse groups. In addition, the reproducibility of the impact on the skin microbiome of the nymphs feeding was striking (Fig. 2J). Indeed, the 4 profiles of mice bitten by nymphs were really similar in terms of composition and proportions, despite technical variability and the fact that there were 4 different mice, from 4 different cages, bitten by 4 different pools of 10 nymphs, and 4 different durations of feeding.

### Impacts on the mouse skin microbiome persist in different experimental conditions

We also checked whether any technical bias in the design of our experiment could explain the dramatic impact of tick feeding on the mouse skin microbiome. Indeed, in this first experiment, the mice bitten by ticks (either adult or nymph ticks) were shaved and a cup was glued on the back of each mouse to maintain the ticks in place and avoid their dispersion. However, the control mice were shaved but did not have a cup. The presence of the cup could impact, among other parameters, the humidity, the temperature, and the oxygen levels of the skin, and hinder the cleaning of the skin by the mice. This could have easily explained a part of the change of the skin microbiome between the control mice (without a cup) and the bitten mice (with a cup). However, the cup could not explain the difference of skin microbiome between mice bitten by adult ticks and mice bitten by nymph ticks (both with a cup) nor explain the transfer of microbiome from the tick to the skin discussed below.

In order to check the impact of the cup on the skin microbiome and confirm the change of skin microbiome due to the tick feeding, we performed a second experiment with new controls and a slightly different protocol of feeding (Fig. 3). In this second experiment, four groups of mice (each mouse placed in an individual cage) were analyzed: two groups of control mice and two groups of mice bitten by ticks. For the control, one group of mice was only shaved and kept for 13 days before being euthanized and sampled, and other groups of mice were shaved and a cup was placed for 3 days before being euthanized and sampled 10 days later.

As illustrated in Fig. 3, there were no significant differences between control mice with or without a cup, both in terms of alpha diversity (Fig. 3A), phylum composition and proportions (Fig. 3B), genus composition and proportions (Fig. 3C), or taxa difference analyzed with LEfSe (Fig. 3D). This demonstrated that the cup did not impact significantly the skin microbiome and that the dramatic difference of skin microbiome observed in Fig. 2 between the control mice and the mice bitten by ticks could not be explained by the presence or absence of a cup.

In addition, this experiment confirms the impact of a tick bite on the skin microbiome (Fig. 3A–C, E–F) and the difference between the skin microbiome of mice bitten either by adult ticks or nymphs (Fig. 3A–C, G). In this second experiment, the overall impact of the tick feeding on the mouse skin microbiome was less dramatic than the one of the first experiment (Fig. 2). Figure 2 used a different protocol (allowing 10 days after tick removal) and ticks were collected at a different time of the year (June for the first experiment of Fig. 2 and April for this second experiment). Common modifications were found in the two experiments (such as a significant increase of *Mycobacterium* genus, *p*=0.018), and some differences were interesting such as the important proportions of *Borrelia/Borreliella* genus appearing in the skin microbiome of all mice bitten with nymphs while being absent in control mice (*p*=0.020).

### Long-term skin microbiome modifications are different between mice bitten by female ticks or by nymphs

In the next experiment, we studied the long-term impact on the skin microbiome of the mice bitten either by female ticks or by nymphs (Fig. 4). Mice were bitten by ticks (either 5 adult female ticks or 10 nymph ticks) for 1, 2, or 3 days then the mice were kept 10 additional days after the removal of the ticks before being euthanized and sampled.

Alpha diversity analysis showed a non-significant trend of reduction of alpha diversity 10 days after the bite in the skin of mice bitten 2 or 3 days by ticks, compared to mice bitten for only one day (Fig. 4A). In terms of taxonomic composition (Fig. 4B), the taxonomic profile was more variable between mice compared to the first two experiments in which mice were euthanized and sampled directly after the removal of the ticks. Thus, it is difficult to assess if the overall taxonomic profile of the skin microbiome 10 days after the tick feeding was differently impacted by the duration of the feeding. However, as illustrated in Fig. 4B, bacteria such as the genus *Borreliella/Borrelia* were clearly present (between 10.9 % and 60.5 % of the reads of the skin biopsy), only in three mice bitten for at least 2 days by ticks. We next compared the skin microbiome of mice bitten by either adult female ticks or nymphs (Fig. 4 C–F). The impact of feeding by female ticks or nymphs on the skin microbiome seems different in terms of alpha diversity (Fig. 4C) but not significant and difficult to interpret due to the small number of samples (2 vs 7 due to female samples excluded for technical reasons). However, the taxonomic composition of the skin microbiome was clearly different between the two mice bitten by female ticks and the seven mice bitten by nymphs as illustrated in the taxonomic barplots at the genus level (Fig. 4D), and the beta diversity PCoA using Bray Curtis distance (*p*=0.047 with PERMANOVA test, n.s. with PERMDISP test for both Bray-Curtis distance, Fig. 4E).

Genera such as *Escherichia-Shigella* were present in an important proportion (27.3 % or 33.6 %) only in the skin microbiome of mice bitten by female ticks, whereas genera such as *Borreliella/Borrelia*, *Gemella*, *Lactobacillus*, *Pseudomonas* or *Streptococcus* were present or increased in the mice bitten by nymphs or both female ticks and nymphs.

### Ticks harbor different microbiome compositions according to their maturity and feeding state

**Fig. 5:**
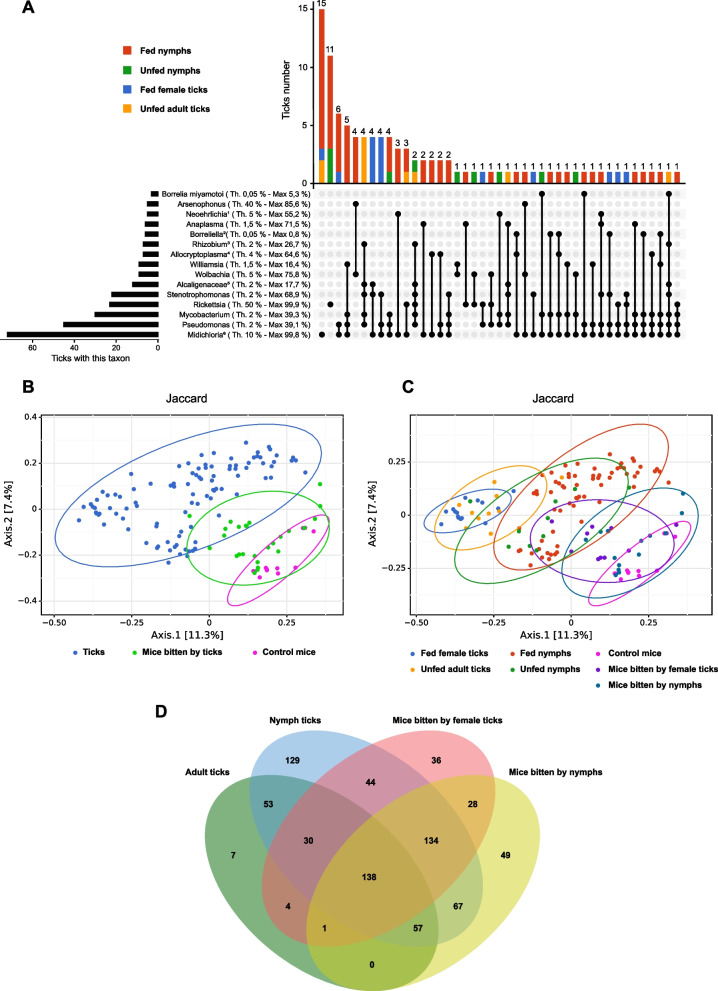
The skin microbiome composition of the mice bitten by ticks gets closer to the microbiome of ticks. **A** Upset plot representation of the presence and co-occurrence of 15 taxa of interest in the ticks. The presence of the taxa is determined using a fixed threshold of total reads % in the sample mentioned in the figure. Th.: fixed threshold used for the corresponding taxa. Max: % of the taxa in the tick which has the highest proportion. Black horizontal barplots represent the number of ticks with % of the corresponding taxa above the fixed threshold. Colored vertical barplots represent the number of ticks with the corresponding combination of taxa above the fixed threshold, separated by fed nymphs (red), unfed nymphs (green), fed female ticks (blue), and unfed adult ticks (yellow). ^1^*Candidatus* Neoehrlichia; ^2^*B. afzelii* or *B. burgdorferi*; ^3^*Allorhizobium-Neorhizobium-Pararhizobium-Rhizobium*; ^4^*Candidatus* Allocryptoplasma; ^5^Multi-affiliated genera of Alcaligenaceae family (*Achromobacter* or *Bordetella*); ^6^*Candidatus* Midichloria. **B** Beta diversity ordination (PCoA) using Jaccard distances of the tick microbiome (all ticks, blue dots) and the skin microbiome of mice bitten by ticks (green dots) and of control mice (pink dots). **C** Beta diversity ordination (PCoA) using Jaccard distances of the tick microbiome separated by maturity and feeding state (blue, yellow, red, and green dots), the skin microbiome of mice bitten by female ticks (dark blue dots) or by nymphs (light blue dots), and the skin microbiome of control mice (pink dots). **D** Venn diagram of the common clusters present in microbiomes of adult ticks (green), nymph ticks (blue), skin of mice bitten by female ticks (pink), and skin of mice bitten by nymphs (yellow). In the Venn diagram, taxa identified as structural zeros by the ANCOM-II (analysis of compositions of microbiome II) preprocessing method were removed prior to plotting the diagram

We analyzed by 16S targeted sequencing the microbiome of 100 whole ticks (without washing the cuticle) either adults or nymphs and either fed or unfed. The unfed ticks (adults and nymphs) were harvested at the same time and the same place as the fed ticks (the ticks used in the experiment of Fig. 4 and Table 3 analyzed after their feeding), during the peak of *Ixodes* activity. As shown on the taxonomic barplot (Supplemental Fig. 3) upset plot (Fig. 5A) and heatmap (Supplemental Fig. 4A and B), ticks can be separated into 5 different groups depending on the predominant taxa of their microbiome profile. As seen in Fig. 5A and Supplemental Figs. 3 and 4, most of the ticks (> 60 ticks, nymphs or adults; fed or unfed) have *Midichloria* (*Candidatus* Midochloria) as the predominant genus; 33 of them have more than 80 % of the reads corresponding to *Midichloria*, 13 ticks more than 95 % of the reads, and up to 99.77 % of the reads for one fed female tick. In 15 ticks, *Midichloria* is the only genus present in the upset plot (Fig. 5A). The second main population of ticks (23 ticks, nymphs or adults; fed or unfed) had mostly reads corresponding to the *Rickettsia* genus (up to 99.78 % of the reads for one of the fed nymph ticks), with sometimes also non-negligible proportions of *Midichloria* (up to 34,62 % of *Midichloria* in one fed female tick with 60.48 % of *Rickettsia*). In 11 ticks, *Rickettsia* is the only genus present in the upset plot (Fig. 5A). The third group corresponds to six ticks (exclusively nymphs fed or unfed) which exhibit mostly DNA from *Wolbachia* (up to 75.79 %) associated with 5 of them, with also an important proportion from *Arsenophonus* (up to 85.57 %). As discussed later, *Wolbachia* and *Arsenophonus* are two genera known to be present in a parasitic wasp of *Ixodes ricinus* and *Ixodiphagus hookeri* [48, 49]. Since the presence of tick parasitic wasp eggs of larvae could induce a biological artifact (*Wolbachia* and *Arsenophonus* do not infect ticks themselves), we performed further analysis (Fig. 5, B–D) on tick microbiome with or without excluding those six ticks and verified that the results were not impacted by those parasitized ticks. Analysis of the 16S sequencing data showed that none of the ticks having at least 6 % of *Wolbachia* possesses proportions of the bacterial pathogen, *Anaplasma*, *Borrelia/Borreliella*, *Neoehrlichia*, or *Ricketsia*, above the technical background levels (data not shown). The fourth group corresponds to ticks with high proportions of *Stenotrophomonas* (up to 53.78 %) associated with lower proportions of other genera, such as for instance *Pseudomonas* or *Candidatus* Midichloria. Finally, the last group is composed of six nymphs which have each a specific predominant taxon. One nymph has 71.54 % of *Anaplasma*, two nymphs have more than 50 % of *Candidatus* Neoehrlichia and three nymphs have between 23.97 % and 64.61 % of *Candidatus* Allocryptoplasma. Five of these six nymphs also presented significant proportions of *Candidatus* Midichloria (between 15.60 % and 44.12 %), but proportions were always inferior to the ones of the above-mentioned genera.

### Skin microbiome composition of the mice bitten by ticks gets closer to the microbiome of ticks

Finally, we compared by beta diversity PCoA analysis (Fig. 5B and C) and Venn diagram (Fig. 5D), the skin microbiome of the mice (bitten or not bitten) from the different experiments and the tick microbiome (adults or nymphs, fed, or unfed). Beta diversity analyses show that the tick microbiome (all ticks: adult or nymphs, fed, or unfed) was clearly different from the skin microbiome of the different control mice not bitten by ticks (Fig 5B, *p*=0.0007 with PERMANOVA test, n.s.with PERMDISP test). However, the skin microbiome of mice bitten by ticks not only are different from the microbiome of control mice (as also observed in Figs. 2, 3 and 4, *p*=0.0045 with PERMANOVA test, n.s. with PERMDISP test), but are also closer to the microbiome of the ticks, confirming that the modification of the skin microbiome after skin feeding observed in Figs. 2, 3 and 4 could result, at least partially, from a transfer of the tick microbiome to the mouse skin (Fig. 5B). In addition, we analyzed the beta diversities of the tick and skin microbiome, by separating ticks by their maturity (adult or nymph), feeding state (fed or unfed), and by separating the skin depending on the maturity of the ticks feeding on the mice (Fig. 5C). Confirming the analyses in Fig. 5A, there is a substantial structuration in terms of beta diversity between female and nymph ticks (*p*=0.0005 with PERMANOVA test, *p*=0.00005.with PERMDISP test for fed females vs fed nymphs; *p*=0.0015 with PERMANOVA test, *p*=0.008 with PERMDISP test for unfed adults vs unfed nymphs), with a shift depending on the feeding state (*p*=0.0005 with PERMANOVA test, n.s.with PERMDISP test for unfed adults vs fed females; *p*=0.0005 with PERMANOVA test, n.s.with PERMDISP test for unfed nymphs vs fed nymphs) (Fig. 5C). This figure confirms that the skin microbiome of mice bitten by ticks is closer to the microbiome of ticks than to the skin microbiome of control mice. Although it is difficult to objectively quantify, in terms of beta diversity it seems that the skin microbiome of mice bitten by adult ticks got closer to the adult tick microbiome and that the skin microbiome of mice bitten by nymphs got closer to the nymph tick microbiome. To analyze this tendency more deeply, we used a Venn Diagram representation of the clusters; after applying ANCOM-II structural zero detection filtering on the data [47], to study the common taxa between the different groups of ticks and skin biopsies (Fig. 5D). In this figure, the bottom barplot first indicates that nymphs have the most diverse microbiome, with 652 different clusters, including 129 clusters specific to the nymphs and 53 clusters shared only with the adult ticks. In contrast, adult ticks only have 290 clusters, with only 7 of them specific to adult ticks. Nymphs have more clusters shared only with the skin of mice bitten by nymphs (67) than clusters shared only with the skin of mice bitten by adults (44). Similarly, adult ticks have more clusters shared only with the skin of mice bitten by adults (4) than clusters shared only with the skin of mice bitten by nymphs (0). This last observation is another clue which shows that not only the skin microbiome of bitten mice got closer to the tick microbiome, but that the skin microbiome of mice bitten by nymphs got closer to the nymph microbiome and that the skin microbiome of mice bitten by adult ticks got closer to the adult tick microbiome.

### Microbiome of nymph is modified by the mice skin microbiome

As described above, the analysis of beta diversity (Fig. 5C) shows a small shift in the beta diversity analysis between the fed and unfed nymphs and between the fed and unfed adult ticks. The barplot of Supplemental Fig. 3 shows that several fed nymphs (marked with an arrow on the barplot of Supplemental Fig. 3) present increased proportions or appearance of several taxa present in the skin microbiome of the mice used in the same experiment (Fig. 3B, D).

*Pseudomonas* genus is present in the skin of all the mice (up to 24.39% of the reads), it has a small prevalence/proportion in unfed nymphs, it is increased in the fed nymphs (up to 29.43 % of the reads, *p*=0.004 with Mann-Whitney test for fed nymph vs unfed nymphs, *p*<0.0001 for fed nymphs with arrows vs unfed nymphs) and is also largely present in adult ticks (up to 39.15%).

*Sphingomonas* genus is present in the skin of 8 of the 10 mice (up to 3.74 % of the reads), is increased in average in the fed nymphs (up to 7.59 % of the reads, n.s. for fed nymphs vs unfed nymphs, *p*=0.031 for fed nymphs with arrows vs unfed nymphs), and is barely detectable in adult ticks.

*Helicobacter* genus (*Helicobacter typhlonius*, *Helicobacter apodemus*, and for one sample *Helicobacter pylori*) is present in the skin of 7 of the 10 mice (up to 4.10 % of the reads), is increased in the nymphs after feeding (up to 7.85 %, n.s. for the fed nymphs vs unfed nymphs, *p*=0.0005 for fed nymphs with arrows vs unfed nymphs), and is absent in adult ticks.

A multi-affiliated genus of the Micrococcaceae family and an unknown genus of the Muribaculaceae family are present in the skin of all mice (up to 3.86% and 11.20 % of the reads respectively), absent or barely detectable in adult ticks and unfed nymphs, and are increased in the fed nymphs (up to 5.50 % and 5.51 % of the reads, respectively, *p*=0.0002 for Microccaceae fed nymphs vs unfed nymphs, *p*<0.0001 for Microccaceae fed nymphs with arrows vs unfed nymphs, *p*=0.038 for Muribaculaceae fed nymph vs unfed nymphs, and *p*=0.0003 for Muribaculaceae fed nymphs with arrows vs unfed nymphs).

### Transmission and dissemination of tick-borne bacteria to mice

We then studied in the different experiments the transmission to the skin, the persistence, and their dissemination to other body sites (blood, heart, and spleen) of tick-borne bacterial pathogens. We used PCR targeting *Borrelia burgdorferi sensu lato* complex (20 species, including *B. burgdorferi*, *B. afzelii*, and *B. garinii*) [8, 50], PCR targeting *Anaplasma phagocytophilum*, and our 16S metagenomic sequencing pipeline.

We analyzed by PCR and by 16S targeted sequencing the presence of pathogens (Table 1) in ticks and mouse skin biopsies used in experiments of Figs. 2 and 3. As mentioned in Table 1, 5 females and 10 nymphs have been fed per mice. Before the blood meal, the positivity of ticks was checked by conventional PCR targeting either *B. burgdorferi* s.l. or *A*. *phagocytophilum*. Nymphs were positive at 15% (3/20) for *B. burgdorferi* s.l. and 10% (2/20) for *A*. *phagocytophilum*. Females were positive at 10% (1/10) for *B. burgdorferi* s.l. and 30% (3/10) for *A*. *phagocytophilum*. After the blood meal (3 days for females and 5 days for nymphs), ticks were removed and tested individually by PCR for these two pathogens. Few ticks in contact with mice did not feed and were not tested. The mouse skin was tested directly by PCR for *B. burgdorferi* s.l. and *A. phagocytophilum*. In parallel, we analyzed the skin biopsy in 16S targeted sequencing (Table 1), to detect and quantify the proportion of DNA of *Borrelia/Borreliella*, *Anaplasma*, and other bacterial genera of interest including potential bacterial pathogens (*Candidatus* Neoehrlichia, *Candidatus* Allocryptoplasma, *Rickettsia*, *Williamsia*), tick symbionts (*Candidatus* Midichloria), and parasitoid wasp symbionts (*Arsenophonus* and *Wolbachia*).

In PCR, only skin biopsies of some mice in contact with ticks at least 4 days were found positive for *Borrelia* or *Anaplasma*. Skin biopsies of mice in contact with ticks less than 4 days (including ticks positive for *Borrelia* or *Anaplasma*) were never positive in PCR (Table 1).

By 16S targeted sequencing, all the bacteria genera studied were totally absent in the skin of control mice, except in three samples which exhibited very low proportions of *Borrelia* DNA (0.1 % of bacterial DNA of *Borrelia*, most likely due to technical inter-sample contamination). In the mice bitten by ticks, the presence in the skin of *Anaplasma* and *Borrelia* found by PCR was confirmed by 16S targeted sequencing. Two other skin samples from mice in contact with *Borrelia*-positive ticks were negative in Borrelia PCR. They possessed small proportions of *Borrelia* DNA in 16S sequencing, but were too close to the background levels measured in negative controls to be conclusive. *Candidatus* Midichloria DNA was found in four mouse skin biopsies, including one with 1.71 % of total reads. *Candidatus* Neoehrlichia DNA was found in two mouse skins in small proportions (≤ 0.27 %). *Candidatus* Allocryptoplasma was found in one skin biopsy with a proportion of 2.13 % of the total reads. *Rickettsia* was present (up to 8.85 %) in the skin of 8 of the 16 mice in contact with ticks, whereas it was absent in the skin of all control mice. *Williamsia* was present (up to 3.39 %) in the skin of 11 of the 16 mice in contact with ticks, whereas it was absent in the skin of all control mice. Finally, *Wolbachia* was present in one skin biopsy (2.71 %).

To test the early dissemination of the bacterial pathogens from the skin, we analyzed different samples (blood, heart, and spleen) by 16S targeted sequencing (Table 2). In the first experiment (Table 1 and Fig. 2) where the skin was directly collected after tick removal from the eight mice, none of the bacterial genera mentioned previously was found at a level above the background, except for *Borrelia miyamotoi* DNA detected in one spleen sample at 0.49 % and in one blood sample at 0.04%.

We then tested the skin, blood, and spleen of the mice used in the third experiment Fig. 4 (Table 3 for potential bacterial dissemination 10 days after tick removal). *Borrelia/Borreliella* was detected in the three sample types. *Borrelia miyamotoi* DNA was detected at 8.31 % in one blood sample and 0.53 % in one spleen sample. *Borreliella* (*B. afzelli* or *B. burgdorferi* s.s*.*) were detected above background levels in 4 skin samples (up to 60.47 % of the total reads) and one spleen sample (7.06 % of the total reads). *Midichloria* (*Candidatus* Midichloria) was found in one blood sample (1.77 %) and one skin biopsy (0.59 %), and a trace close to the background (potentially a technical contamination) was detected in one spleen sample (0.003 %). *Rickettsia* was detected in one blood sample (1.47 %), one skin biopsy (0.12%), and one spleen sample (0.01%). Finally, *Williamsia* was detected in one skin biopsy (2.09 %) and one spleen sample (0.77%). All other bacteria of interest (*Anaplasma*, *Arsenophonus*, *Candidatus* Neoehrlichia, *Candidatus* Allocryptoplasma, and *Wolbachia*) had strictly null values in all the samples of this experiment of bacterial dissemination.

## Discussion

### Alteration of the mouse skin microbiome after tick bites

Skin microbiome represents an extensive field of study in humans, with numerous published studies related to health and dermo-cosmetic applications [51]. However, skin microbiome is much less studied in mice, and only a handful of studies have been published [52–58]. Those studies describe a rich microbiome in the mouse skin which differs significantly between different reports. This variability between studies is expected and explained by biological and technical biases already present in classical microbiome analyses in the gut (such as different strains of mice, different diets, different animal house, different cages, interindividual and temporal variabilities, different analysis protocols…) [59, 60]. This variability also presents a challenge for the interpretation of the skin microbiome composition observed in our control mice. However, several of the main bacterial genera in the skin of the control mice of our study (*Acinetobacter*, *Bacillus*, *Bacteroides*, *Enterococcus*, *Micrococcus*, *Moraxella*, *Pseudomonas*, *Sphingomonas*, *Staphylococcus*, *Stenotrophomonas*, *Streptococcus*, *Streptomyces*) are also reported in one or several published studies which seems to confirm that the basal skin microbiome in our study corresponds to a classical microbiome for laboratory mice. According to the literature on both human and mouse microbiomes, as well as in our own experience, the skin microbiome is considered as rich both in terms of quantity and diversity (one of the richest microbiomes in the body after the gut). Thus, the dramatic impact of tick bite on the skin microbiome of the mice is really striking, especially considering three parameters observed in our study: (1) the entire skin microbiome is completely modified at the site of the tick bite. After the tick bite, the alpha and beta diversities of the skin microbiome were completely altered. The vast majority of the taxa which represent most of the reads in the control commensal skin microbiome disappear or become negligible and are replaced by taxa which were originally absent or slightly present. (2) The alteration of the microbiome is mostly dependent on the maturity of the ticks, with a more pronounced impact of nymph bite in all our experiments compared to adult ticks. (3) The impact of the tick bite on the skin microbiome, especially involving nymphs, is extremely reproducible both in terms of alpha diversity and taxonomic composition. As explained above, microbiome profiles are usually very variable between individuals due to well-known biological and technical biases. In the first experiment described in Fig. 2, each sample belongs to a different mouse, housed in a different cage, and bitten by different wild type ticks, for different durations of feeding. Nevertheless, the altered skin microbiome of the different samples is nearly identical both in terms of alpha diversity and taxonomic composition especially when bitten by nymphs, with reproducibility close to one expected for technical replicates.

Since most of the taxa of the commensal skin microbiome of the control mice were replaced after the tick bite by bacterial taxa absent or negligible originally, one of the main hypotheses is to assume that the new altered microbiome consists, at least partly, of bacterial taxa transmitted from the ticks to the mice skin. Bacterial taxa overrepresented in the skin after the tick bites could also derive from the original skin microbiome (taxa with really low proportions in normal conditions), from translocation of bacteria from other parts of the mice (mouth, feces …) or from external sources (cage, food, air …).

In any case, the dramatic modification of the skin microbiome is likely to be triggered or allowed by the modification of the mouse skin physiology caused by the tick bite, in terms of composition, metabolism [61–63], immunity, and inflammation [22, 62–65]. Some of these modifications induced by the tick bite, and in particular the tick saliva, are discussed more in detail in the last part of the discussion.

To be noted that several bacterial families dramatically reduced by the tick bite, such as Lachnospiraceae or Muribaculaceae are bacteria usually reported as beneficial for health in mice or humans (in skin or gut) [66–69]. Conversely, most genera increased in the skin after the tick bite, such as *Erwinia*, *Mycobacterium*, *Rhodococcus*, *Stenotrophomonas*, or *Streptomyces* are reported in humans as skin pathogens [70–76] or negatively correlated with healthy skin [77, 78]. Overall, not only the dramatic shift of microbiome triggered by the tick bite most likely affects by itself the normal skin physiology, but the composition of the new microbiome suggests a particularly negative impact on the health of the skin.

### The microbiome, symbionts and pathogens within the tick

To better understand the interaction between the skin and tick microbiomes, we studied in the third experiment the microbiome of 100 wild-type *I. Ricinus* ticks, unfed and fed on mice

Ticks harbor a specific microbiome required for nutritional provisioning and regulation of their innate immune system [16, 25, 79]. Some are maternally inherited microbes, transmitted transovarially [28], and others are acquired from the environment either in the vegetation during the free phase in the humus or from the vertebrate host during their blood meal [16]. In our study, ticks were not washed before microbiome analysis, to assess the whole tick microbiome in the condition of the field where ticks are covered by microorganisms from environmental origin [16, 23]. We consider that the microorganisms at the surface of the ticks (cuticle) are part of the natural tick microbiome and should play a role during the natural tick bite to the vertebrate host and the modification of the host microbiome. In addition, a previous study has demonstrated the technical impact (DNA denaturation) on the tick gut microbiome of several methods of surface sterilization [23].

With the development of metagenomics, different tick genera have been investigated for their microbiome and the list of identified microorganisms has been set [18, 80–82]. We focused our study on *I. ricinus*, a major vector of different pathogenic microorganisms in Europe. It can harbor a great variety of microorganisms including pathogenic bacteria (*B. burgdorferi* s.l., *Anaplasma phagocytophilum*, *Rickettsia helvetica*, *Borrelia miyamoto*i, *Neoerhlichia mikurensis*,…), parasites (*Babesia* spp.), and viruses (Tick-Borne Encephalitis) [11, 83]. The microbiome composition of *I. ricinus* and its variability according to geography and between life stages is more and more studied [18, 56, 84]. However, potential exchanges of bacterial taxa with the vertebrate host are still poorly investigated. The most common bacterial genera described in the literature in *I. ricinus* (*Midichloria*, *Rickettsia*, *Wolbachia*, *Arsenophus*, *Mycobacterium*, and *Stenotrophomonas*) were observed in our study with the exception of the *Spiroplasma* genus [25, 56, 85]. This latter bacterium is a common maternally inherited endosymbiont of ticks [86], but its prevalence typically shows significant variation in infection patterns among populations of *Ixodes* tick species. Indeed, *Spiroplasma* prevalence ranges from 0 to 30% depending on tick populations and species [25]. Furthermore, it has been shown recently that in field-collected ticks, a negative association exists between *Rickettsia* and *Spiroplasma* [56]. The ticks collected in the present study harbor a high level of *Rickettsia* that might explain the absence of *Spiroplasma* in our data.

### Transmission and dissemination of tick-borne bacteria to the skin

While the transmission of well-recognized pathogens such as members of *B. burgdorferi* s.l. complex [87, 88] and *A. phagocytophilum* [89] is well studied, the potential co-transmission to the host of other microbes is poorly documented for ticks. In a recent study compared by metatranscriptomics, the bacterial and viral RNA found in the skin biopsies of 14 humans bitten by ticks with the RNA found in the ticks removed from the patients [90]. Authors cited 4 bacterial genera (*Pseudomonas*, *Acinetobacter*, *Corynebacterium*, and *Cutibacterium/Propionibacterium*), found in both the patient skins and the ticks, and suggested that the bacteria could be transmitted from the tick to the skin. However, the absence of control in the study, and the fact that the four genera are common bacteria found in healthy human skin [51] (three of them were actually found present in the skin of the control mice not bitten by ticks in our study), does not allow a definitive statement. Another recent study has shown in mouse models that the host skin microbiome might be a factor determining the transmission of rickettsial pathogens from ticks [91]. A comparative analysis of the microbiome of fed and unfed *I. persulcatus* ticks and of the blood of rats bitten by these ticks has shown that 237 bacterial genera were common to the three sample types suggesting a significant transmission of tick microorganisms to the rat blood [92]. *Midichloria* DNA was found in several skin, blood, and spleen samples in tick-bitten mice of our study. The endosymbiont *Midichloria mitochondrii* is present in ovaries and in salivary glands of *I. ricinus* [29, 82]. Previous studies have also detected the presence of *Midichloria* DNA in the blood of mammals or human patients exposed to ticks, which can further produce antibodies against the bacteria [93–96]. In such case, since *Midichloria* DNA can be released by the tick saliva, the detection of *Midichloria* DNA in vertebrates is usually interpreted just as a marker of tick bite and not as the presence of viable bacteria or infection [94]. Furthermore, the absence of clinical signs or symptoms in humans tested positive for Candidatus *Midichloria* DNA seems to confirm that this tick endosymbiont is probably not a pathogen [96].

In the transmission experiment, some bacterial genera were particularly well represented like *Mycobacterium* in nymph-bitten skin and *Stenotrophomonas* in nymph and female-bitten skin. *Stenotrophomonas* is a Gram-Proteobacteria present in the soil and in plants. It has regularly been detected in different tick genera such as *Haemaphysalis* [97], in *Ixodes* [18, 21, 85] and in *Rhipicephalus* [80]*.* Similarly, *Mycobacterium* and *Pseudomonas* are also well-conserved bacteria in the tick microbiome [85].

*Williamsia* spp., a mycolic acid–containing actinomycetes of the suborder Corynebacterineae, has been described recently as an opportunistic bacteria in humans [98]. This bacterium has been identified in different mouse skins, mainly after a female tick bite, up to 3.39% of the total reads.

*Wolbachia* and *Arsenophonus* have also been described in *I. ricinus* as a consequence of parasitism by *Ixodiphagus hookeri* parasitoid wasps [49]. These two bacteria are maternally inherited endosymbionts of arthropods and filarial nematodes. Both are the most common bacteria detected in not only arthropods, including wasps, butterflies, and spiders, but also mosquitoes and bedbugs [99]. While infections have not been detected either in humans or other vertebrates, meta-analysis estimates that ~40% of all arthropod species are infected with *Wolbachia* [100]. Interestingly, in our study, *Wolbachia* was detected once in the mouse skin and it was not present in mouse control skin suggesting that *Wolbachia* DNA traces could be released by the tick saliva, as observed for *Candidatus* Midichloria.

Interestingly, in our study, among the 9 ticks with at least a small proportion of *Wolbachia* (≥ 6 % of total reads), none had proportions of the bacterial pathogens *Anaplasma*, *Borrelia/Borreliella*, *Neoehrlichia*, or *Rickettsia* above the technical background levels. On the other hand, 32 ticks without *Wolbachia* had levels of those pathogens above the background level. This observation seems similar to published data which shows that in different mosquito species (such as *Aedes aegypti* or *Anopheles gambiae*), the presence of *Wolbachia* inhibits the infection of the mosquito by various human pathogens such as the Dengue virus, the Chikungunya virus, and the malaria parasite *Plasmodium falciparum* [6, 7, 101, 102]*.* This negative association could be an unlikely coincidence due to a random distribution and has to be further investigated in other studies. If confirmed, this association could open new strategies to control tick-borne human diseases. Concerning the transmission of pathogens and their detection at day 0 after the tick removal, we were only able to detect *Anaplasma* and *Borrelia/Borreliella* when the ticks have fed for at least 4 days, whatever the technique conventional PCR or 16S sequencing. The absence of detection before the fourth day can also be linked to the low proportions of these bacteria in the skin during the first day. Indeed, it is generally admitted that bacteria are inoculated by the ticks to the vertebrate host after at least 24 h of feeding [103].

To study the long-term impact of tick feeding in skin microbiome and pathogen dissemination in mice, we chose to study the skin and tissue microbiomes 10 days after the removal of the ticks from the mice, since this time point corresponds to a peak of pathogen multiplication in the mouse skin [40]. The impact of tick feeding on the skin microbiome is still present 10 days after the feeding, but to a lesser and less reproducible extent than directly after the removal of the ticks. However, as expected from the literature, the prevalence and read proportions of *Borrelia*/*Borreliella* are widely increased 10 days after the tick removal, especially in the skin of mice bitten by nymph ticks. Indeed, *Borrelia* is barely present in the skin of mice bitten by adult ticks in both experiments, but present in significant proportions in 4/4 skin biopsies of the mice bitten by nymphs in the second experiment (between 2.62 % and 28.25 % of the reads) and in 3/10 skin biopsies of the mice bitten by nymphs in the third experiment (between 10.87 % and 60.47 % of the reads). This is correlated with the higher prevalence of *Borrelia* measured by PCR in the nymphs compared to adult ticks from experiments 1 and 2. It should be noted that this prevalence was measured after the feeding and includes potential transmission of *Borrelia* between ticks during the feeding. In addition, in the second and third experiments, the experimental protocol might explain this difference in positivity between mice bitten by nymphs and female ticks. Indeed, females were left for 3 days on mice and nymphs for 5 days. In natural conditions, feeding lasts up to 10 days for female ticks, and they have at least one more blood feeding compared to the nymphs. In field studies, the prevalence of *Borrelia* is usually greater in female ticks than in nymphs; thus, a female tick has more opportunities to transmit the pathogen to the host [104].

In the two mice with the highest levels of *Borrelia/Borreliella* DNA in their skin biopsies, *Borrelia/Borreliella* DNA is also detected in two spleen biopsies and one blood sample. Lower levels of *Borrelia* were also detected in one spleen biopsy of the first experiment just after the removal of the ticks. Overall, *B. afzelli*, *B. burgdorferi* s.s., and *B. miyamotoi* DNAs were detected in the different mouse tissues, but *B. miyamotoi* was never detected in the skin and was present only in the spleen and blood. It is not surprising since this *Borrelia* belongs to the relapsing fever group and induces a high bacteremia in the vertebrate host and is reported to be more present in the blood than in the skin [105]. We cannot ascertain whether the *Borrelia/Borreliella* DNA corresponds to living/dormant bacteria or free/intracellular bacterial DNA. However, the proportion of reads (8.31 % in the blood and up to 7.06 % in the spleen) is considered high, in comparison to the richness of the blood and tissue bacterial DNA load and diversity [34, 37]. In addition, one mouse has a high level of *Borreliella* in its skin and high level of *Borrelia miyamotoi* in its spleen and blood. Thus, the high level of DNA in the spleen and blood, in this case, does not seem to result from DNA translocated from skin-living bacteria. According to the literature [105], this difference could indeed correspond to a specific tropism of the *Borrelia/Borreliella* species in the body.

Unlike *Borrelia/Borreliella*, other pathogenic or tick-specific bacteria (*Anaplasma*, *Midichloria*, *Neoerlichia*, *Allocryptoplasma*, *Rickettsia*, *Williamsia*, and *Wolbiacha*) seem to be transmitted to the mice skin without obvious difference between nymphs or adult ticks (Table 1). In the transmission experiment (collection at day 0 after tick removal), the prevalence of those bacteria in the skin appeared higher than in the dissemination experiments (10 days after tick removal), but the small number of positive samples combined with the fact that the different experiments used ticks harvested at different times does not allow a definitive statement. In the dissemination experiment, however, significant levels of *Midichloria* DNA and *Rickettsia* DNA were found in two different blood samples, and *Williamsia* DNA was found in one spleen sample. Again, whether this DNA could belong to living bacteria remains to be investigated. *Arsenophonus* DNA was found in none of the skin, spleen, or blood samples in any experiment.

### Alteration of the tick microbiome after feeding

In addition to the striking impact of the tick bite on the mouse skin microbiome, to a lesser extent, the tick microbiome is also modified after the feeding on the mouse. Fed nymphs present important proportions of taxa (such as *Pseudomonas*, *Sphingomonas*, *Helicobacter*, genera of Micrococcaceae and Muribaculaceae families) present in the mice of the same experiment (control or bitten by these ticks). These taxa are absent or present in significantly lower proportions in unfed nymphs and have likely been translocated from the mouse skin to the nymph during the feeding. The microbiome of female ticks also appears to be impacted by the feeding, but unlike the nymphs, we did not observe obvious profiles of translocation of mouse skin bacterial taxa in the female ticks. This can be explained by a shorter blood meal for female ticks or a less mature microbiome more sensitive to modifications in nymphs. *Pseudomonas* genus, present in the mouse skin and increased in fed nymphs, is present in both fed and unfed adults. The presence of *Pseudomonas* in adult mice could be the result of its transmission from animal hosts during a previous blood meal. However, since *Pseudomonas* is also a genus of bacteria widely present in the environment, soil, and water [106], its origin from previous feeding is uncertain.

### Microbiome, pathogens and vector competence

As discussed above, the microbiomes of fed and unfed ticks display a very high interindividual variability both in terms of the presence of pathogenic bacteria and in terms of overall taxonomic profiles. However, the present study also points out the impressive reproducibility of the alteration in the bitten mouse skin microbiome. The consistence of the skin microbiome in the mice bitten by ticks with a variable microbiome suggests that it results from biological mechanisms which are not random but controlled. The tick and its microbiome seem to alter the mouse commensal microbiome and immune system to allow the establishment of a new skin microbiome, likely needed for the tick feeding and which could play a major role in pathogen transmission to the host.

It is known that modifications of the gut and salivary gland microbiomes, two important organs for tick-borne pathogen development, can impact the dynamics of pathogen transmission by triggering the host immune system or modifying organ integrity [22]. In *I. scapularis*, changes in the gut microbiome affect the integrity of the peritrophic matrix produced during the blood meal which subsequently affects the establishment of *Borrelia* pathogens [1]. Similarly, disruption of the gut microbiome of *Dermacentor andersoni* by antibiotic treatment modifies the tick-vector competence for *Anaplasma marginale* [107]. A recent analysis performed with field-collected ticks showed that some associations exist between bacteria of the tick microbiome and transmitted pathogens. For example, a positive association might exist between *M. mitochondrii*, members of the *B. burgdorferi* s.l. complex and *N. mikurensis* [56]. *Midichloria* might increase the tick survival by providing essential nutrients [25]. The role of bacterial symbionts in vector competence has been particularly well documented in mosquitoes. As discussed above, *Wolbachia* for example, alters the vector competence of different species of mosquito for different pathogens. Our results seem to suggest a similar negative association in ticks between several human pathogens and *Wolbachia*.

The human microbiome modulates the inflammation at the skin interface [108, 109]. Therefore, the arthropod microbiome has been logically investigated as a potential actor during the process of vector-borne pathogen transmission. Recent data [33] on *Leishmania donovani* transmission confirmed this hypothesis. These parasites are co-inoculated with sandfly microbiomes leading to inflammasome activation and secretion of IL-1 beta in the vertebrate host. In the transmission of tick-borne pathogens, very few studies have been performed to elucidate the potential transmission of gut or salivary gland microbiome during the process of the tick bite via exosomes [61]. Interestingly, some of these tick symbionts have been shown to be transmitted during the tick bite process, due to their presence in salivary glands, such as *Coxiella*-like endosymbiont, a pathogen found in a human skin biopsy in Europe [110]. In the present study, we have evidenced that at least part of the tick microbiome is transferred to the mouse skin during the tick bite. This replacement of the host microbiome might facilitate the transmission of pathogens by downregulating the potential effect of host-microbiome on inflammation. This particular aspect deserves further investigation.

## Conclusion

In this study, we analyzed the impact of blood feeding on tick and mouse skin microbiomes in different experimental conditions. Host skin microbiome at the bite site was deeply impacted by tick bite, to an extent which suggests both a role in the tick feeding and a potential important impact in the skin physiopathology. Most of the commensal bacteria present in the skin of control mice are replaced during the blood-feeding process by bacteria also present in the ticks. Our main hypothesis is that the commensal skin microbiome is, at least partially, replaced by bacteria from the tick microbiome. However, this hypothesis is based on the comparison of the different microbiomes and future studies will be required to confirm it. In addition, we have seen that several pathogens can be transmitted to the skin microbiome and disseminated to the blood or spleen of the mouse. Skin microbiome alteration and *Borrelia* transmission were different depending on the maturity of feeding ticks (nymphs versus adult female ticks) and appeared more dramatic with nymphs. At this point, we are cautious in the interpretation, since nymphs were left long enough to complete their blood meal unlike female ticks.

Concerning the tick microbiome, we showed diversified taxonomic profiles which are modified by the feeding. This modification could either be caused by a translocation of bacteria from the host skin and/or the variation of proportions of taxa already present in the ticks. This modification of the tick microbiome by the blood meal on the vertebrate host was more pronounced in nymph ticks.

Several questions were raised by this study and will have to be studied in the future:

- What is the extent of the skin microbiome alteration outside the site of the tick bite?
- How does this profound microbiome modification impact the health and normal skin function of the vertebrate host?
- What it the role of microbiome modifications (both in the host and in the tick) in terms of tick escape of the host immune system and in terms of transmission of the pathogen to the host?
- Is the microbiome alteration observed in murine hosts reproducible in human hosts, and what are the consequences in terms of skin immunity and tick-borne infection in humans?
- Does a negative correlation exist between the presence of *Wolbachia* and human pathogens in ticks as observed in mosquitoes?

### Supplementary Information

**Additional file 1:** **Fig. S1.** A, B, Comparison of beta diversities by hierarchical clustering analysis of the 16S rRNA gene sequencing data using Generalized UniFrac (GUniFrac, alpha=0.4) dissimilarity distances in the ticks samples (A) or the mouse skin samples (B) and their respective negative controls (H_2_O-Ext). C,D Venn diagram of the common clusters present in microbiomes of the ticks samples (C) or the mouse skin samples (D) and their respective negative controls (H_2_O-Ext). In the Venn diagram, taxa identified as structural zeros by ANCOM-II (Analysis of Compositions of Microbiome II) preprocessing method were removed prior to plot the diagram. H_2_O-Ext: molecular grade water extracted, amplified and sequenced at the same time as the corresponding samples.

**Additional file 2:** **Fig. S2.** Barplots of relative proportions in the skin biopsies of all genera above 1% on average in all samples grouped by duration of feeding (barplots similar to those of Figure 2.I which display only top 20 genera).

**Additional file 3:** **Fig. S3.** Barplots of relative taxa proportions in the ticks of top 20 genera grouped by maturity and feeding state of the ticks. Arrow marks fed nymphs with taxonomic profiles displaying clearly increased proportions or appearance (compared to unfed nymphs) of several taxa present in the skin microbiome of the mice used in the same experiment.

**Additional file 4:** **Fig. S4.** A, B. Heatmaps of the proportions (% of total reads) of the 15 taxa of interest of Fig. 5A in using a linear scale color key (A) or log scale color key (B). Fed nymphs (red), unfed nymphs (green), fed female ticks (blue) and unfed adult ticks (yellow). ^1^*Candidatus* Neoehrlichia; ^2^*B. afzelii* or *B. burgdorferi;*^3^*Allorhizobium-Neorhizobium-Pararhizobium-Rhizobium;*^4^*Candidatus* Allocryptoplasma; ^5^Multi-affiliated genera of Alcaligenaceae family (*Achromobacter* or *Bordetella*); ^6^*Candidatus* Midichloria.

## Data Availability

The sequencing data for this study have been deposited in the European Nucleotide Archive (ENA) at EMBL-EBI under accession number PRJEB61985 https://www.ebi.ac.uk/ena/browser/view/PRJEB61985.
