# Supplementary figures and images for: Cross-alteration of murine skin and tick microbiome concomitant with pathogen transmission after Ixodes ricinus bite
Source: Microbiome. 2023 Nov 11;11:250. doi: 10.1186/s40168-023-01696-7 (PMC10638774; doi:10.1186/s40168-023-01696-7)

Supplemental Figure 1

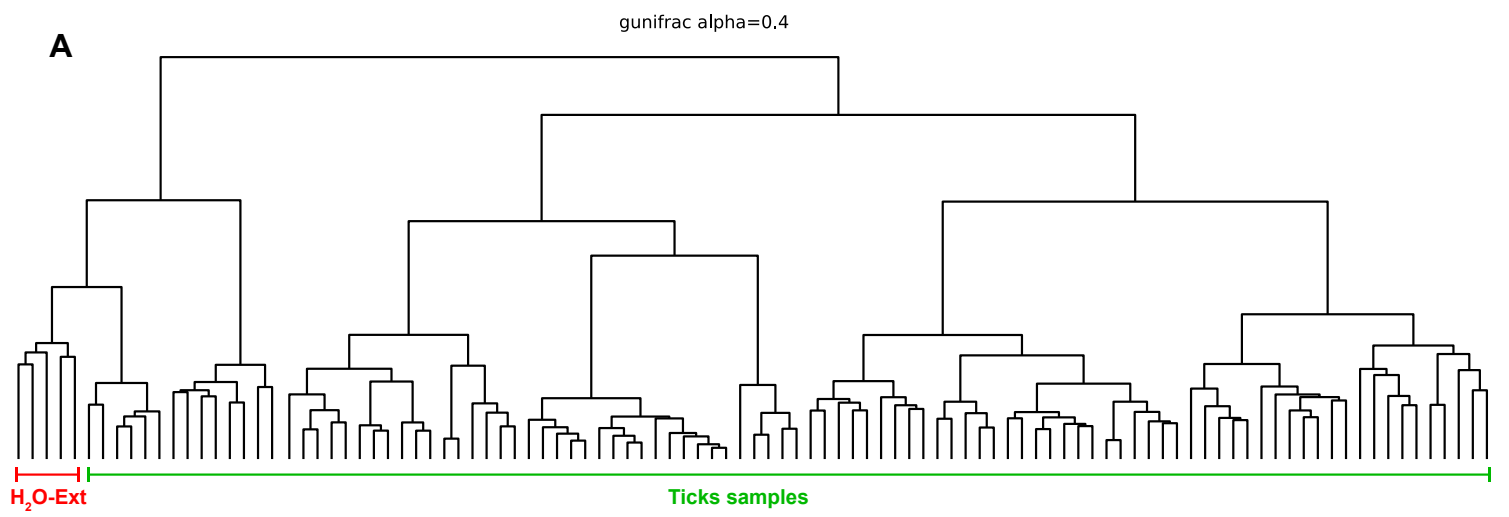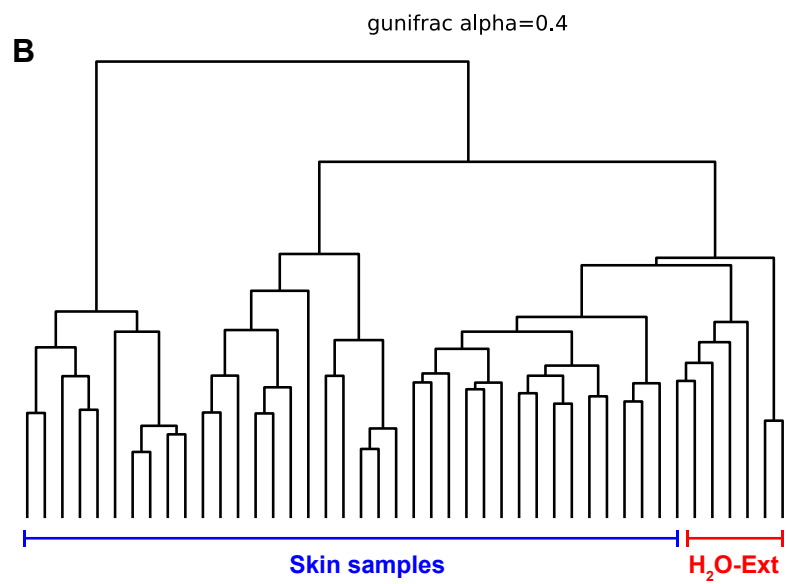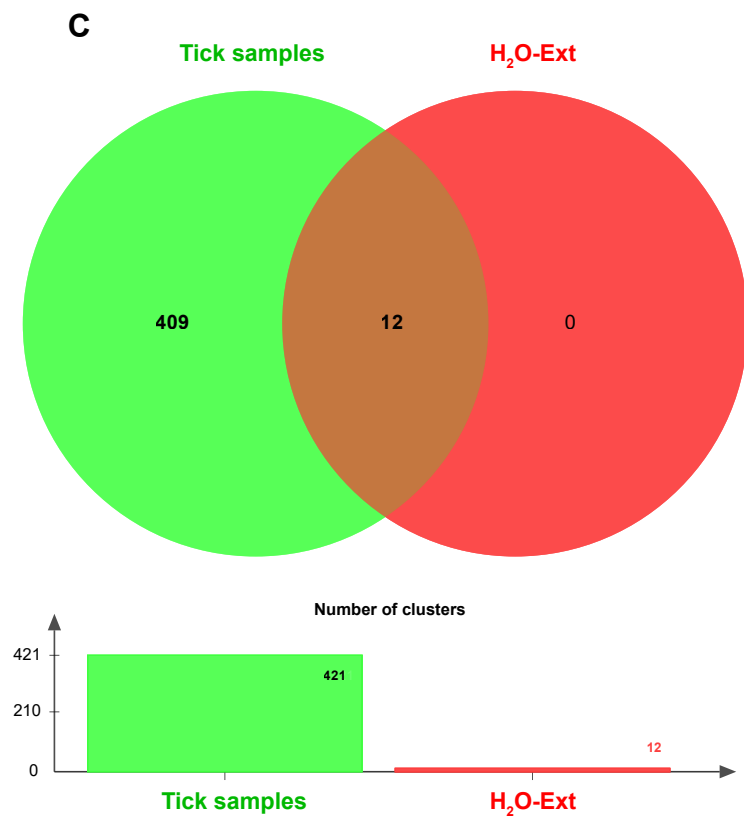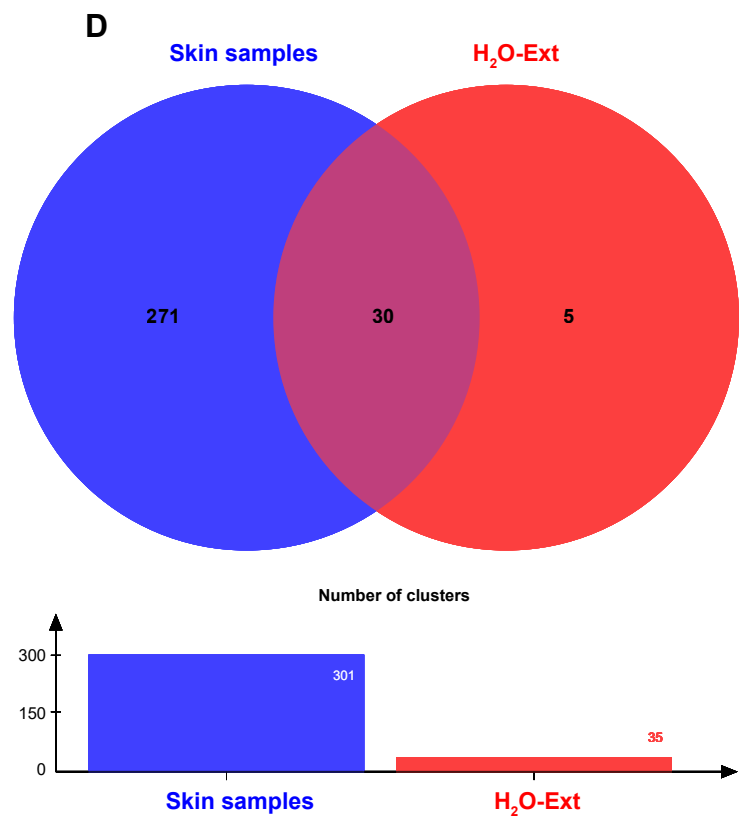

Supplement: Supplementary file 2 — Additional file 1: Fig. S1. A, B, Comparison of beta diversities by hierarchical clustering analysis of the 16S rRNA gene sequencing data using Generalized UniFrac (GUniFrac, alpha=0.4) dissimilarity distances in the ticks samples (A) or the mouse skin samples (B) and their respective negative controls (H2O-Ext). C,D Venn diagram of the common clusters present in microbiomes of the ticks samples (C) or the mouse skin samples (D) and their respective negative controls (H2O-Ext). In the Venn diagram, taxa identified as structural zeros by ANCOM-II (Analysis of Compositions of Microbiome II) preprocessing method were removed prior to plot the diagram. H2O-Ext: molecular grade water extracted, amplified and sequenced at the same time as the corresponding samples. [file 40168_2023_1696_MOESM1_ESM.pdf]

Supplemental Figure 2

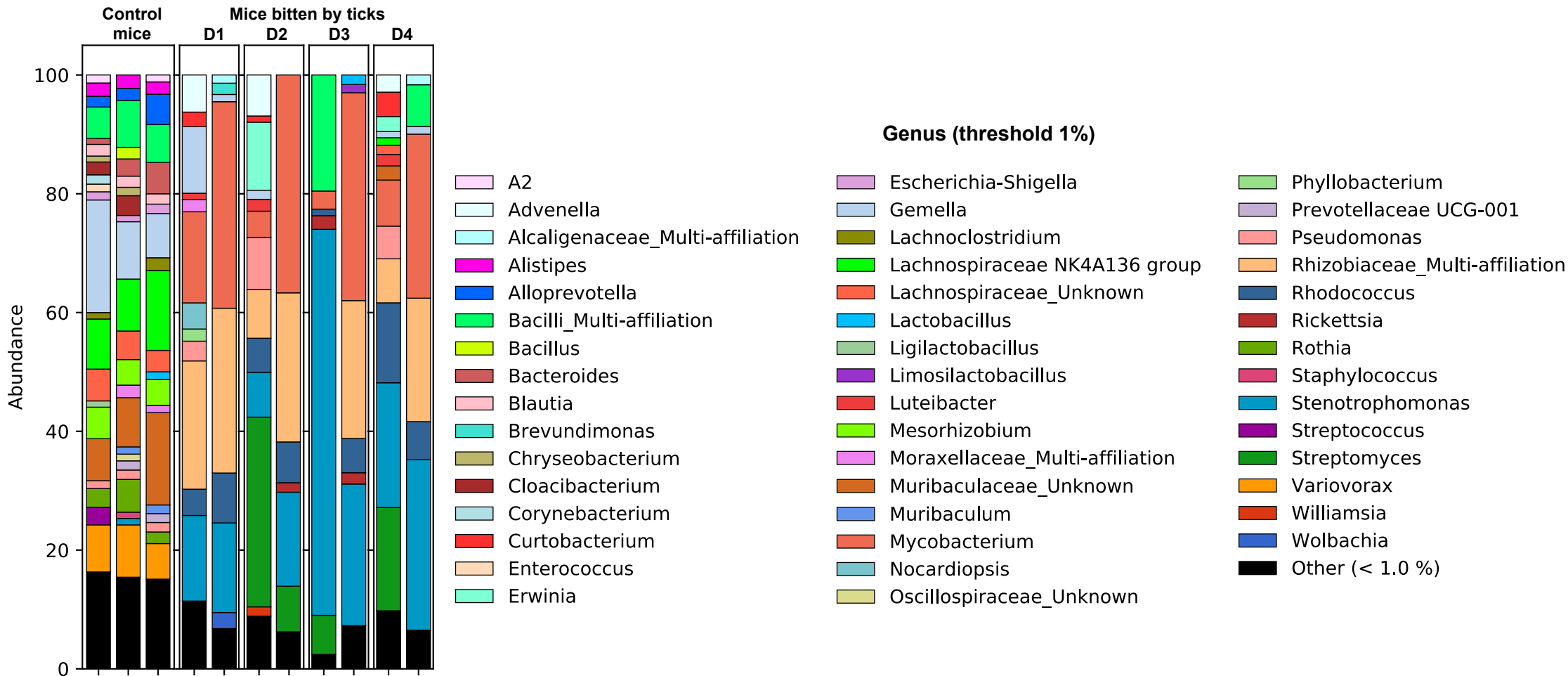

Supplement: Supplementary file 3 — Additional file 2: Fig. S2. Barplots of relative proportions in the skin biopsies of all genera above 1% on average in all samples grouped by duration of feeding (barplots similar to those of Figure 2.I which display only top 20 genera). [file 40168_2023_1696_MOESM2_ESM.pdf]

Supplemental Figure 3

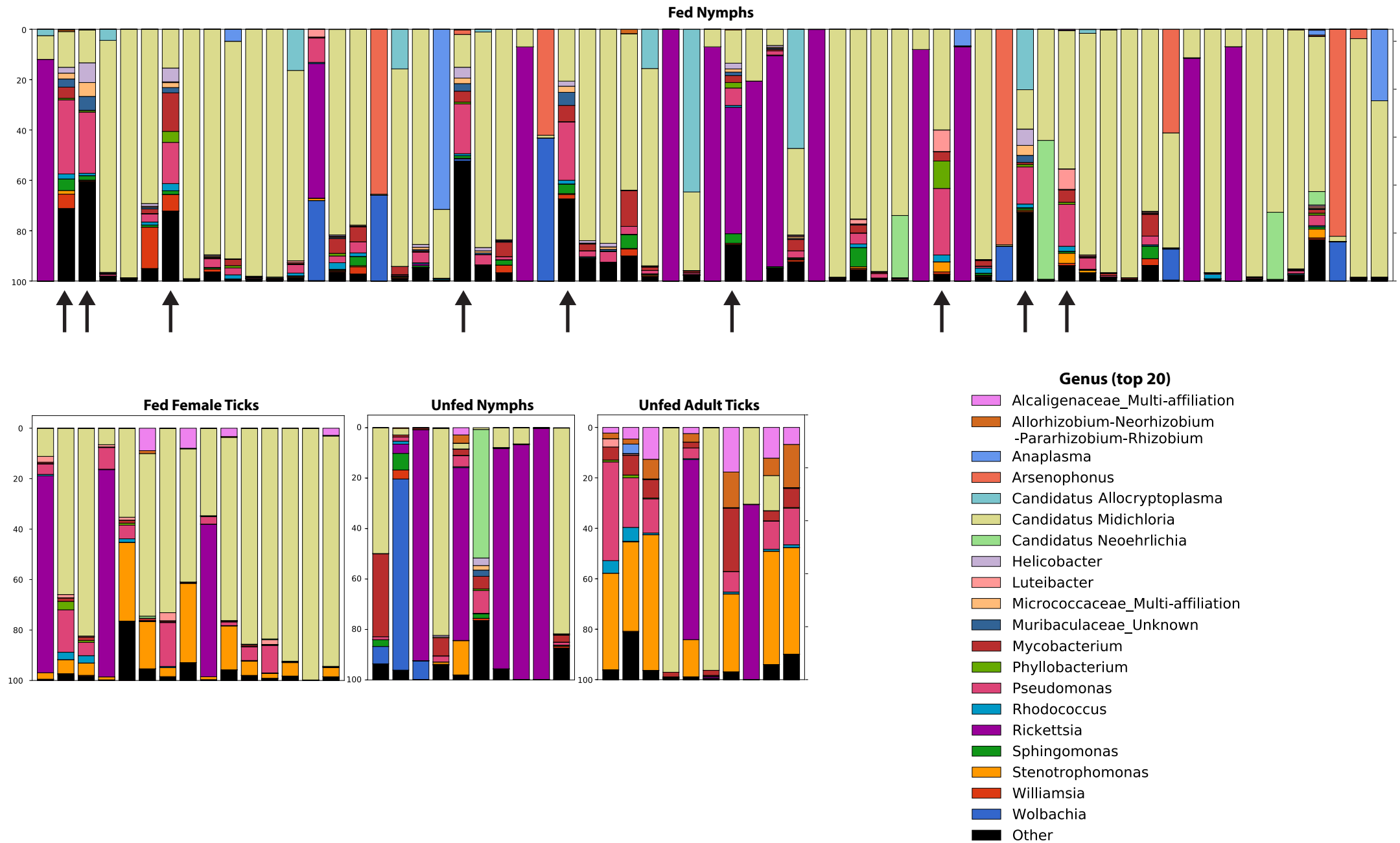

Supplement: Supplementary file 4 — Additional file 3: Fig. S3. Barplots of relative taxa proportions in the ticks of top 20 genera grouped by maturity and feeding state of the ticks. Arrow marks fed nymphs with taxonomic profiles displaying clearly increased proportions or appearance (compared to unfed nymphs) of several taxa present in the skin microbiome of the mice used in the same experiment. [file 40168_2023_1696_MOESM3_ESM.pdf]

Supplemental Figure 4

A - Linear scale

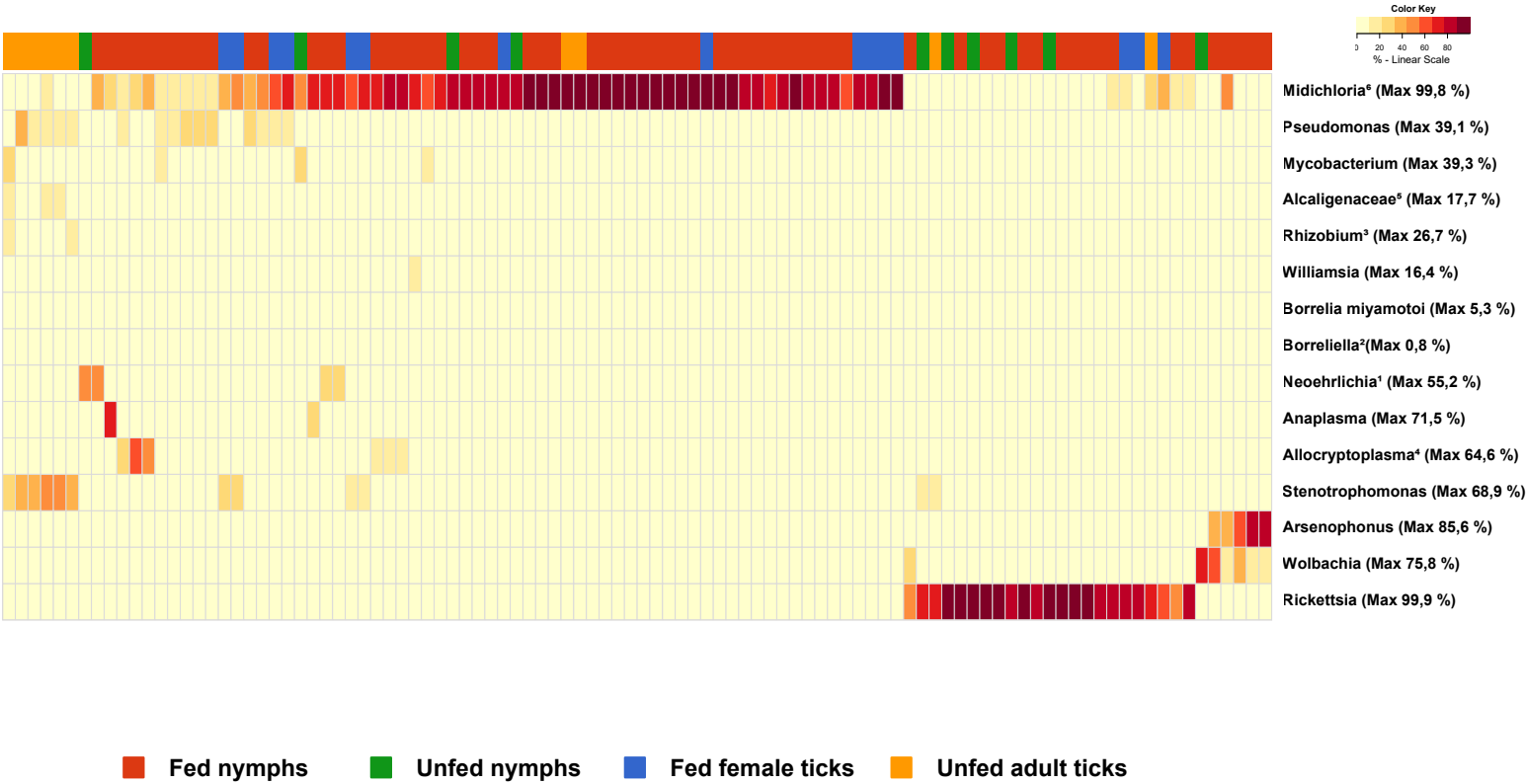

B - Log scale

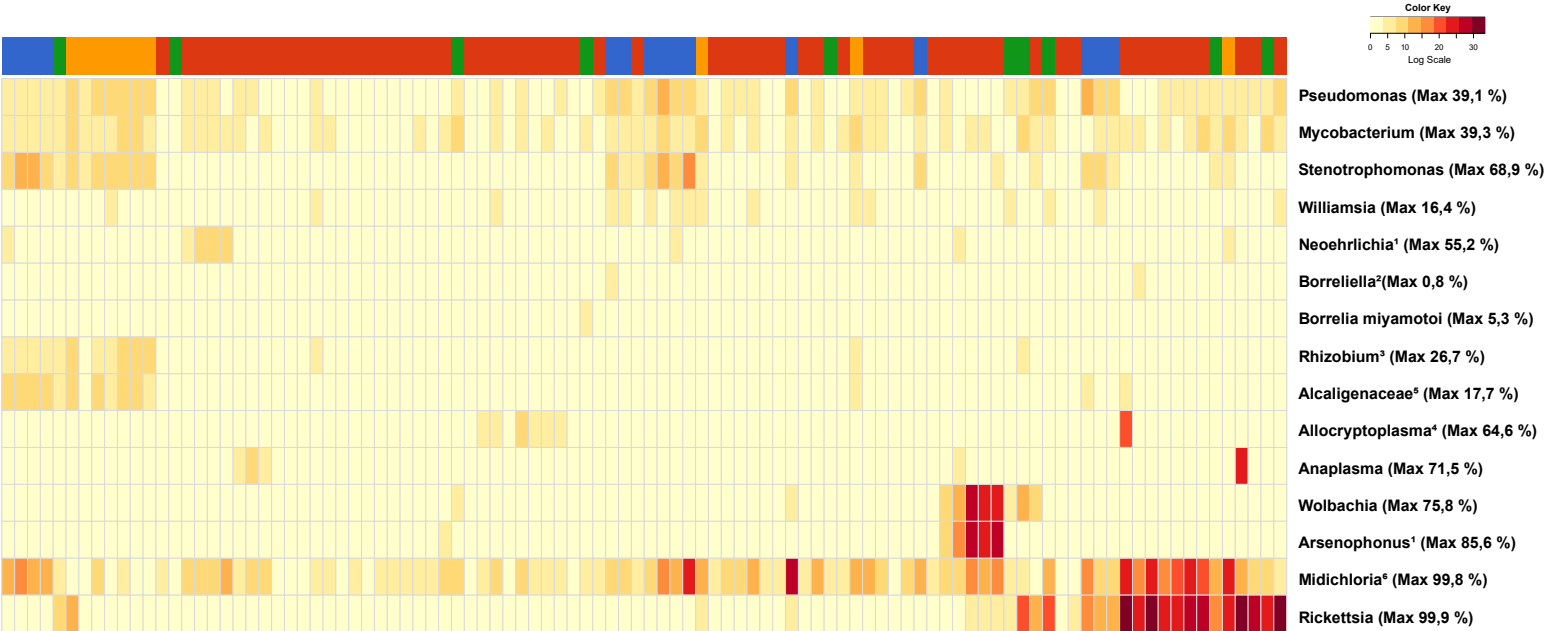

Supplement: Supplementary file 5 — Additional file 4: Fig. S4. A, B. Heatmaps of the proportions (% of total reads) of the 15 taxa of interest of Fig. 5A in using a linear scale color key (A) or log scale color key (B). Fed nymphs (red), unfed nymphs (green), fed female ticks (blue) and unfed adult ticks (yellow). 1Candidatus Neoehrlichia; 2B. afzelii or B. burgdorferi;3Allorhizobium-Neorhizobium-Pararhizobium-Rhizobium;4Candidatus Allocryptoplasma; 5Multi-affiliated genera of Alcaligenaceae family (Achromobacter or Bordetella); 6Candidatus Midichloria. [file 40168_2023_1696_MOESM4_ESM.pdf]
